# The health impact of smokeless tobacco products: a systematic review

**DOI:** 10.1186/s12954-021-00557-6

**Published:** 2021-12-04

**Authors:** C. Hajat, E. Stein, L. Ramstrom, S. Shantikumar, R. Polosa

**Affiliations:** 1 Public Health Institute, UAE University, Abu Dhabi, 15551 UAE; 2 Independent Researcher, New York, USA; 3 Independent Researcher, Institute for Tobacco Studies, Stockholm, Sweden; 4 Warwick Medical School, University of Warwick, Coventry, UK; 5 Center of Excellence for the Acceleration of HArm Reduction (CoEHAR), University of Catania, Catania, Italy; 6 Department of Clinical and Experimental Medicine, University of Catania, Catania, Italy

**Keywords:** Tobacco harm reduction, Smokeless tobacco, Snus, Snuff, Moist snuff, Smoking, Tobacco, Cardiovascular disease, Cancer, Mortality, Respiratory disease, Mental health

## Abstract

**Introduction:**

The objective was to systematically review studies on health outcomes from smokeless tobacco (SLT) products.

**Methods:**

We analysed published literature on the health outcomes from SLT use between 01/01/2015 to 01/02/2020, following Preferred Reporting Items for Systematic Reviews and Meta-Analyses (PRISMA) protocol using PubMed, Embase, Scopus, and Google Scholar.

**Results:**

Of 53 studies included, six were global, 32 from Asia, Middle East and Africa (AMEA), nine from USA and six from Europe. ‘Poor’-rated studies predominated (23;43%), in particular, for global (4;66%) and AMEA (16;50%). Health outcomes differed between SLT-products and regions; those in AMEA were associated with higher mortality (overall, cancer, Coronary heart disease (CHD), respiratory but not cardiovascular disease (CVD)), and morbidity (CVD, oral and head and neck cancers), with odds ratios up to 38.7. European studies showed no excess mortality (overall, CVD, from cancers) or morbidity (ischemic heart disease (IHD), stroke, oral, head and neck, pancreatic or colon cancers) from several meta-analyses; single studies reported elevated risk of rectal cancer and respiratory disorders. Pooled study data showed protection against developing Parkinson’s disease. US studies showed mixed results for mortality (raised overall, CHD, cancer and smoking-related cancer mortality; no excess risk of respiratory or CVD mortality). Morbidity outcomes were also mixed, with some evidence of increased IHD, stroke and cancer risk (oral, head and neck). No studies reported on switching from cigarettes to SLT-products.

**Conclusion:**

Our review demonstrates stark differences between different SLT-products in different regions, ranging from zero harm from European snus to greatly increased health risks in AMEA. The literature on the safety profile for SLT-products for harm reduction is incomplete and potentially misinforming policy and regulation.

**Supplementary Information:**

The online version contains supplementary material available at 10.1186/s12954-021-00557-6.

## Introduction

The use of SLT-products exceeds that of all other forms of tobacco use in some parts of the world. The prevalence of SLT-product use in men is 30% in India, 6% in Iceland [1], and 20% in Sweden [2]. SLT is rising in parts of Europe and some have attributed its use to the concomitant reduction in smoking prevalence [3–5].

There are numerous types of SLT-products available globally which differ markedly in terms of their preparation, method of use and toxicity.[6] Key features of some of the most common SLT-products are detailed in “Appendix 1”. Although there has been no clear consensus on safety profiles of SLT-products, it is generally accepted that they pose a lower health risk than cigarettes. Despite the many differences described above, SLT-products are often regarded together as a single product and safety concerns have resulted in varying regulations and bans on sales and use globally. The objective of this systematic review was to identify, narratively synthesize, assess the strength and quality of evidence, and critically appraise studies that report health outcomes associated with use of different SLT-products in different regions of the world.

## Methods

We conducted a systematic review of published literature on the health impact of SLT-products between January 1, 2015, and February 1, 2020. SLT-products included all types including snus, chewing tobacco, snuff and other products included in Table 1 (“Appendix 1”). For the purpose of this review, we reported findings according to three geographical regions, which best align with different types of SLT-products consumed, namely Europe (EU), the Americas (USA), and SE Asia, Eastern Mediterranean and Africa (AMEA) regions. The study followed PRISMA guidelines for reporting systematic reviews [7]. We included health outcomes of new onset or control of disease end-points. We did not include other health outcomes such as short-term physiological changes which do not necessarily manifest as disease or quality of life or in vitro effects.

**Table 1:** Types of SLT products by World Health Organization region

Tobacco product	WHO region					
	AFR	AMR	EMR	EUR	SEAR	WPR
**Oral use**						
Betel quid with tobacco			X		X	X
*Chimó*		X				
Creamy snuff					X	
Dry snuff	X	X		X		
*Gul*					X	
*Gudhaku*					X	
*Gutka*					X	
*Iq'mik*		X				
*Khaini*					X	
*Khiwam*					X	
Loose leaf		X		X		
*Maras*				X		
*Mawa*					X	
*Mishri*					X	
Moist snuff		X		X		
*Naswar*	X		X	X		
Plug chewing tobacco		X				
Red tooth powder					X	
*Shammah*			X	X		
Tobacco chewing gum						X
Tobacco tablet		X				
*Toombak*	X					
*Tuibur*					X	
Twist/roll chewing tobacco		X				
*Zarda*			X		X	
**Nasal use**						
Dry snuff	X		X	X	X	
Liquid snuff	X					

African Region (AFR), Region of the Americas (AMR), South-East Asian Region (SEAR), European Region (EUR), Eastern Mediterranean Region (EMR), Western Pacific Region (WPR)

### Search strategy and eligibility criteria

A literature search was conducted between October 1, 2019, and February 26, 2020, using the databases PubMed, Embase, Scopus, and Google Scholar using medical subject headings.

There were two domains: one for SLT-products and one for health outcomes, specifically CVD, cancer, respiratory, mortality and ‘other’ health outcomes. Search terms included “Smokeless tobacco” OR “smokeless tobacco product” OR “chewing tobacco”OR “reduced risk tobacco”OR “non-cigarette tobacco” OR “snus” OR

“snuff” AND “health outcome” OR “morbidity” OR “mortality” OR “cancer”OR “cardiovascular disease”OR “chronic obstruct pulmonary disease” OR “COPD” OR “CVD” OR “acute myocardial infarction”OR “stroke” OR “cardiovascular” OR “cerebrovascular”OR “health effects”OR “adverse” OR “effects” OR “respiratory”.

Search results were filtered to include English language, human studies and studies published from 01/01/2015 until 01/02/2020, in order to capture current product types and their changing pattern of use. The health outcomes of interest such as mortality, cancer and CVD, can take many years to develop and manifest and would still have been captured from use of historical SLT products. The references of relevant reviews were manually searched for additional eligible citations.

Titles, abstracts and full texts of the search results were sequentially screened by two reviewers independently for inclusion, using the eligibility criteria below, with disagreements resolved via blind review by a third reviewer.

**Fig. 1:**
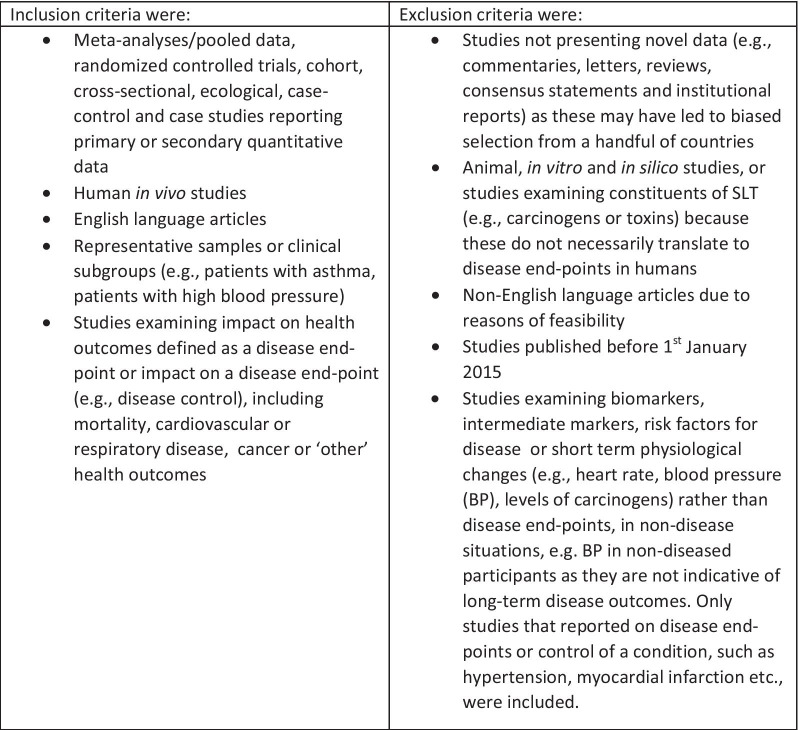
.

**Fig. 2:**
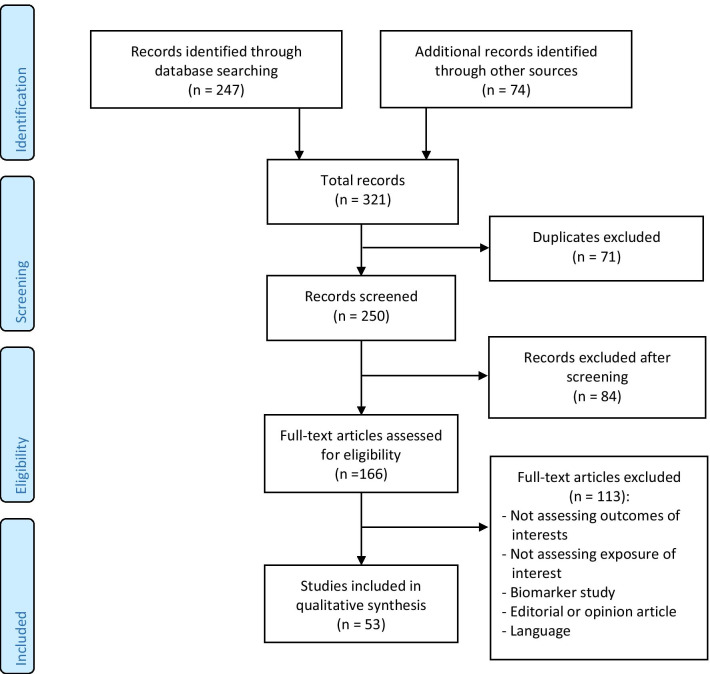
.

Figure 1 shows the inclusion and exclusion criteria used. Reasons for excluding studies are shown in Fig. 2.

### Data extraction and quality assessment

For included studies, data were extracted including author, year, country, aim, study design, sample size, participants and relevant findings such as effect sizes and nature of impact on health outcomes. Studies were categorized by region including global, AMEA, USA and EU. A level of evidence category was assigned using the Oxford Centre for Evidence Based Medicine framework [8] and a similar approach used to categorise methodological quality as “good”, “fair” or “poor” utilizing the National Institutes for Health (NIH) Quality Assessment Tools [9]. The NIH quality assessment tools include features to assess risk of bias, such as selection and reporting bias, with a “good” rating reflecting a low risk of bias, and a “poor” rating suggesting a high risk of bias. Data extraction and synthesis was performed by two reviewers independently with blind assessment by a third reviewer for cases with rater disagreement. Findings of all studies were independently reviewed, coded and compared between studies to identify relationships and themes.

We considered a meta-analysis of studies included in our review to be inappropriate, partly due to the common methodological flaws and the vast heterogeneity between studies. As such no statistical testing was required, only narrative reporting of study findings.

## Results

Of the 53 studies included, six included global data, 32 were exclusively from AMEA, nine exclusively from USA, and six exclusively from Europe. The number of studies by study design and health outcomes are shown in Table 2.

**Table 2:** Number of studies by health outcome and study design

	Mortality	Morbidity			Total
		Cardiovascular	Cancer	Other	
*Global*					
Meta-analysis/Pooled data	4	3	2	0	9
RCT	0	0	0	0	0
Cohort	0	0	0	0	0
Cross-sectional	0	0	0	0	0
Case control	0	0	0	0	0
Case series/report	0	0	0	0	0
Sub-total	4	3	2	0	9
*AMEA*					
Meta-analysis/Pooled data	0	0	4	0	4
RCT	0	0	0	0	0
Cohort	1	1	0	0	2
Cross-sectional	0	4	2	4	10
Case control	1	0	15	0	16
Case series/report	0	0	2	0	2
Sub-total	2	5	23	4	34
*Europe*					
Meta-analysis/Pooled data	1	0	2	1	4
RCT	0	0	0	0	0
Cohort	1	0	0	1	2
Cross-sectional	0	0	0	1	1
Case–control	0	0	0	0	0
Case series/report	0	0	0	0	0
Sub-total	2	0	2	3	7
*US*					
Meta-analysis/Pooled data	1	0	1	0	2
RCT	0	0	0	0	0
Cohort	3	0	0	0	3
Cross-sectional	0	1	0	2	3
Case control	0	0	0	0	0
Case report	0	0	0	0	0
Sub-total	4	1	1	2	8
Total	12	9	28	9	58

Totals for each health outcome and region may include duplication studies that examined more than one health outcome

All six global studies were meta-analyses or of pooled data. Studies from AMEA were predominantly case–control designs (16; 50%) and hospital-based, followed by cross-sectional (8; 25%). In Europe, the commonest study design was meta-analyses (4; 57%) and cohort (4; 28%). In the USA, cross-sectional (3; 38%) and cohort (2; 28%) were the most common study designs. Cancer was the most common outcome, comprising more than two-thirds (23; 72%) of AMEA studies. Mortality was also reported commonly across all regions.

Table 3 summarizes the quality ratings assigned to studies by health outcome, with inter-rater agreement on 49 out of 53 (92%) for quality and level of evidence categoriese. A ‘poor’ rating was commonest (23;43%), followed by fair (21;39%); then good (9;17%). Global (4;66%) and AMEA studies (16;50%) had greater ‘poor’-rated studies. ‘Good’ ratings were given to 33% (2) of global studies, 28% (2) of Europe studies, 6% (2) of AMEA studies and 38% (3) of US studies.

**Table 3:** Quality Ratings Assigned to Studies by Outcome

Health outcome	Good quality	Fair quality	Poor quality
*Global*			
Mortality	1	0	3
Morbidity	2	0	3
Sub-total	3	0	6
*AMEA*			
Mortality	0	1	1
Morbidity	2	12	16
Sub-total	2	13	17
*Europe*			
Mortality	0	2	0
Morbidity	2	3	05
Sub-total	2	5	0
*US*			
Mortality	3	1	0
Morbidity	0	2	2
Sub-total	3	3	2
Total	10	22	26

Totals may reflect duplicated studies that examined more than one health outcome. Quality assigned as “good”, “fair” or “poor” utilizing NIH Quality Assessment Tools [8].

Two studies reported on benefits from SLT-product use; a cross-sectional study on hypertension and a meta-analysis on Parkinson’s disease in Europe.

Table 4 provides a summary of study design, key outcomes, level of evidence, and quality rating for the included studies by region. Additional file 1: Table 5 provides more detailed findings of each study.

**Table 4:** Summary of studies, level of evidence, and quality for health outcomes from SLT by region

Author	Subjects/studies	Outcomes	Impact on health outcome	Level of evidence*	Quality rating
*Global*					
Mortality					
Gupta [10]	14 studies	IHD Mortality, stroke	Fatal stroke: OR = 1.27; 95% CI 1.15–1.39	3A	Poor
Siddiqi [13]	32 studies	DALYs and mortality	1.7 million, DALYs lost and 62,283 deaths due to cancers of mouth, pharynx and oesophagus	3A	Poor
Sinha [12]	16 studies	Mortality due to all cause, cancer mortality	All-cause mortality: OR = 1.33; 95% CI 1.11–1.34 All cancer mortality: OR = 1.31; 95% CI 1.16–1.47 IHD mortality: OR = 1.10; 95% CI 1.04–1.17 Stroke mortality: OR = 1.37; 95% CI 1.24–1.51	3A	Poor
Vidyasagaran [11]	19 studies	IHD mortality, stroke mortality	IHD deaths: OR = 1.15; 95% CI 1.01–1.30 Stroke deaths: OR = 1.39; 95% CI 1.29–1.49	3A	Good
*Morbidity*					
Cancer					
Asthana [52]	37 studies	Oral cancer	Oral cancer: OR = 3.52; 95% CI 2.75–4.51	3A	Poor
Siddiqi [13]	32 studies	Oral, pharyngeal, esophageal cancers	Mouth cancers: OR = 3.43; 95% CI 2.26–5.19 Pharynx cancer: OR = 2.23; 95% CI 1.55–3.20 Esophageal cancers: OR = 2.17; 95% CI 1.70–2.78	3A	Poor
Cardiovascular					
Gupta [10]	14 studies	IHD Mortality, stroke	Stroke: OR = 1.18; 95% CI 1.04–1.32 Fatal stroke: OR = 1.27; 95% CI 1.15–1.39 Nonfatal stroke: OR = 1.03; 95% CI 0.91–1.14	3A	Poor
Gupta [9]	20 studies	CHD	CHD: OR = 1.05; 95% CI 0.96–1.15	2A	Good
Vidyasagaran [11]	19 studies	IHD	IHD: OR = 1.14; 95% CI 0.92–1.42 Stroke: OR = 1.01; 95% CI 0.90–1.13	3A	Good
*AMEA*					
Mortality					
Etemadi [14]	50,045	Overall mortality, IHD mortality, CVA mortality, cancer mortality	Mortality: HR = 1.16; 95% CI 1.01–1.34 Cancer mortality: HR = 1.40; 95% CI 1.01–1.95 IHD mortality: HR = 1.32; 95% CI 1.05–1.67 CVA mortality: HR = 1.06; 95% CI 0.74–1.53 Respiratory mortality: HR = 1.73; 95% CI 0.94–3.19	2B	Fair
Gupta [10]	14 studies	IHD mortality, stroke	Fatal stroke: OR = 1.35; 95% CI 1.18–1.51	3A	Poor
Sinha [12]	16 studies	Mortality, all cancer, UADT cancer, stomach cancer, cervical cancer, IHD, stroke	All-cause mortality: OR = 1.25; 95% CI 1.08–1.44 All cancer mortality: OR = 1.46; 95% CI 1.26–1.68 Stroke mortality: OR = 1.37; 95% CI 1.14–1.64	3A	Poor
Vidyasagaran [11]	19 studies	IHD	IHD mortality: OR = 1.05; 95% CI .076–1.47	3A	Good
Gajalakshmi [15]	22,460 cases, 429,306 controls	All-cause mortality, cancer deaths, respiratory deaths, stroke deaths (verbal autopsy)	All-cause mortality: RR = 1.3; 95% CI 1.2–1.4 Respiratory diseases combined: RR = 1.5; 95% CI 1.4–1.7 Respiratory tuberculosis: RR = 1.7; 95% CI 1.5–1.9 Cancers (all sites combined): RR = 1.5; 95% CI 1.4–1.7 Stroke: RR = 1.4; 95% CI 1.2–1.6	3B	Poor
*Morbidity*					
Cancer					
Gholap [34]	Ecological analysis	Head and neck cancer	(r = 0.53) oropharynx cancer incidence	2C	Fair
Nair [32]	747	Squamous cell carcinoma	No HR/OR reported	2C	Poor
Asthana [52]	37 studies	Oral cancer	Oral cancer in Southeast Asia: OR = 4.44; 95% CI 3.51–5.61 Oral cancer in Eastern Mediterranean:OR = 1.28; 95% CI 1.04–1.56	3A	Poor
Khan [20]	6 studies	Squamous cell carcinoma	Oral cancer: OR = 11.8; 95% CI 11.4–25.3; I2 = 67%	3A	Good
Prasad [78]	22 studies	Six cancers	Oral cancer: OR = 6.6 (95% CI 5.2–8.4) Larynx: cancer OR = 1.42 (95% CI 0.69–2.90) Lung cancer: OR = 2.15 (95% CI 1.22–3.78) Esophagus cancer: OR = 3.46 (95% CI 1.95–5.72) Oropharynx cancer: not significant Hypopharynx cancer: not significant	3A	Fair
Quadri [22]	6 studies	Oral cancer	Oral cancer: OR = 38.74; 95% CI 19.50–76.96	3A	Poor
Siddiqi [13]	32 studies	Oral, pharyngeal, oesophageal cancers	Mouth cancers in India: OR = 5.12; 95% CI 3.27–8.02 Mount cancers in Pakistan: OR = 8.81; 95% CI 3.14–24.69 Pharynx cancer in India: OR = 2.60; 95% CI 1.76–3.85 Oesophageal cancers in India: OR = 2.57; 95% CI 2.20–3.00 Oesophageal cancers in Pakistan: OR = 8.20; 95% CI 1.45–27.47	3A	Poor
Sinha [21]	25 studies	Cancers	Oral cancer: OR = 5.67; 95% CI 3.83–8.40 Pharyngeal cancer: OR = 2.69; 95% CI 2.28–3.17 Oesophageal cancer: OR = 3.17; 95% CI 2.76–3.63	3A	Poor
Alharbi [24]	70 cases, 140 controls	Squamous cell carcinoma of oral cavity	Shammah: OR = 33.01; 95% CI 3.22–39.88 Shisha: OR = 3.96; 95% CI 0.24–63 Shammah and shisha: OR = 35.03; 95% CI 11.50–65.66	3B	Poor
Awan [25]	134 cases, 134 controls	Oral cancer	Gutka: OR = 5.54; 95% CI 2.83–10.83; p < 0.001 Chewing tobacco: OR = 5.32; 95% CI 1.14–24.77; p = 0.033	3B	Fair
Chang [35]	549 cases, 601 controls	Oral cavity, oropharynx, hypopharynx and larynx cancers	Head and neck cancer: OR = 2.39; 95% CI 1.77–3.23	3B	Fair
Gupta [26]	187 cases, 240 controls	Squamous cell carcinoma	OR = 8.51; 95% CI 4.90–14.77	3B	Fair
Hassanin [27]	196	Oral squamous cell carcinoma	OR = 3.8; 95% CI 1.7–8.6	3B	Poor
Kadashetti [30]	100 cases, 100 controls	Oral cancer	OR = 2.8; 95% CI 1.2–7.0	3B	Fair
Khan [28]	84 cases, 174 controls	Oral cancer	OR = 21.0; 95% CI 6.1–72	3B	Poor
Khan [31]	90 cases, 120 controls	Oral cavity cancer	OR = 4.71; 95% CI 2.53–8.74	3B	Poor
Merchant [79]	79 cases, 143 controls	Oral squamous cell carcinoma	OR = 7.39; 95% CI 1.01–38.11	3B	Fair
Mohite [37]	217 cases, 217 controls	Breast cancer	OR = 2.35; 95% CI 1.01–5.51	3B	Poor
Nair [39]	100 cases, 200 controls	Colorectal cancer	OR = 1.53; 95% CI 0.58–4.0	3B	Poor
Quadri [23]	48 cases, 96 controls	Oral cancer	OR = 29.30; 95% CI 10.33–83.13	3B	Fair
Rajbongshi [36]	100 cases, 100 controls	Breast cancer	OR = 2.35; 95% CI 1.3–4.15	3B	Poor
Shah [38]	70 cases, 140 controls	Gastric cancer	OR = 4.37; 95% CI 1.92–9.95	3B	Fair
Sajad[80]	35 year-old male	Squamous cell carcinoma	OSCC in buccal mucosa and the reason for the same was exclusive unilateral tobacco chewing habit, with placement of the tobacco in the right mucobuccal fold	4	Fair
Mahapatra [29]	134 cases, 268 controls	oral cancer	OR = 6.0; 95% CI 2.6–15.5	4	Poor
Soni [33]	100 cases	oral cancer	Significant difference in oral cancer among patients who exclusively chewed tobacco than non-users of tobacco	4	Poor
Cardiovascular					
Gupta [9]	20 studies	CHD	CHD: OR = 1.02; 95% CI 0.86–1 Non-fatal CHD: OR = 1.10; 95% CI 1.00–1.20 Fatal CHD: OR = 1.03; 95% CI 0.86–1.19	2A	Good
Ahwal [19]	90	Hypertension, blood fasting blood glucose, diabetes, dyslipidemia	Dyslipidemia: OR = 6.37; 95% CI 1.4–27.3 Hypertension: OR = 6.97; 95% CI 1.7–28.0	2C	Poor
Anand [17]	4038	Diabetes, asthma, hypertension	Risk of diabetes and hypertension not statistically significant among self-reported exclusive SLT users compared to exclusive smokers	2C	Fair
Bhatt[81]	314	Systolic BP, diastolic BP	Systolic hypertension associated with quantity of SLT use (B = 0.389 (SE = 0.131); p = 0.003), but diastolic hypertension was not (B = 0.122 (SE = 0.087); p = 0.160)	2C	Good
Mishra [18]	36	Latent CHD	No difference in CHD between those who exclusively smoke and those who use exclusive SLT	2C	Poor
Gupta [10]	14 studies	IHD Mortality, stroke	Strok: OR = 1.35; 95% CI 1.18–1.51 Fatal stroke: OR = 1.35; 95% CI 1.18–1.51	3A	Poor
Vidyasagaran [11]	19 studies	IHD	IHD: OR = 1.40; 95% CI 1.01–1.95 IHD death: OR = 1.05; 95% CI .076–1.47	3A	Good
Behera [16]	423	stroke and MI	Stroke: OR = 3.71; 95% CI 1.57–9.05 MI: OR = 2.34; 95% CI 1.10–5.40	4	Poor
Other					
Anand [17]	4038	Diabetes, asthma, hypertension, chronic lung disease	Chronic lung disease: OR = 0.64; 95% CI 0.45–0.91	2C	Fair
Ahwal [19]	90	Hypertension, blood glucose, diabetes, dyslipidemia	There was no difference in the prevalence of obesity (BMI > = 25) among SLT users, smokers and non-tobacco users (p = 0.393)	2C	Poor
Mahapatra [29]	512	Oral health	Gingival bleeding: OR = 1.710; 95% CI 1.2–2.43 Periodontal pockets: OR = 1.715; 95% CI 1.19–2.48 Loss of attachment: OR = 2.393; 95% CI 1.55–3.69	2C	Fair
Mathew [82]	800	Reproductive health	Obstetrical problems: OR = 4.1; 95% CI 2.54–6.64 Neonatal problems: OR = 2.78; 95% CI 1.59–4.87	2C	Poor
*US*					
Mortality					
Fisher [41]	210,090 and 154,286	All-cause and disease-specific (cancer, cardiovascular) mortality	Mortality (NHIS): HR = 1.03; 95% CI .83–1.29; (NLMS): HR = 0.82; 95% CI 0.59–1.13) Malignant neoplasms of: Trachea, bronchus, and lung: HR = 2.98; 95% CI 0.91–9.76 Digestive organs: HR = 1.01; 95% CI 0.32–3.20 Esophagus: HR = 2.44; 95% CI .31–19.1 Pancreas: HR = 1.36; 95% CI .19–9.98 Ggenitourinary system: HR = 0.51; 95% CI 0.007–3.78 Heart disease (NLMS): HR = 1.07; 95% CI 0.65–1.75; (NHIS): HR = 1.20; 95% CI 0.91–1.58 Heart failure (NLMS): HR = 1.13; 95% CI 0.28–4.62; (NHIS): HR = 2.75; 95% CI 1.55–4.89 IHD (NLMS): HR = 0.95; 95% CI 0.49–1.83; (NHIS): HR = 1.06; 95% CI 0.75–1.49	2A	Good
Inoue-Choi [45]	65,335	Cancer-specific mortality	Mortality: HR = 1.36; 95% CI 1.17–1.59 CHD mortality: HR = 1.63; 95% CI 1.27–2.09 Cancer mortality: HR = 1.48; 95% CI 1.04–2.12 Smoking-related cancer mortality: HR = 1.76; 95% CI 1.07–2.90	2B	Good
Rodu [43]	46,104	Mortality	Younger (40–59 years)/all-cause mortality: HR = 1.44; 95% CI 1.12–1.84	2B	Good
Timberlake [44]	349,282	Mortality from all causes, all cancers, CHD, cerebrovascular disease and digestive system cancers	All-cause mortality: HR = 1.01; 95% CI 0.93–1.10 All cancers: HR = 0.99; 95% CI 0.82–1.21 Cerebrovascular disease: HR = 0.92; 95% CI 0.67–1.27 Death from CHD: HR = 1.25; 95% CI 1.05–1.46	2B	Fair
Sinha [12]	16 studies	Mortality due to all cause, all cancer, UADT cancer, stomach cancer, cervical cancer, IHD, stroke	All-cause mortality: OR = 1.17; 95% CI 1.12–1.22 All cancer mortality: OR = 1.14; 95% CI 1.01–1.29 Stroke mortality: OR = 1.44; 95% CI 1.30–1.59 IHD mortality: OR = 1.16; 95% CI 1.05–1.28	3A	Poor
Vidyasagaran [11]	19 studies	IHD	IHD death: OR = 1.03; 95% CI 0.83–1.27	3A	Good
*Morbidity*					
Cancer					
Asthana [52]	37 studies	Oral cancer	OR = 4.72; 95% CI 0.66–33.62	3A	Poor
Siddiqi [13]	32 studies	Oral, pharyngeal, oesophageal cancers	Mouth cancers: OR = 0.95; 95% CI 0.7–1.2 Pharynx cancer: OR = 1.59; 95% CI 0.84–3.01 Oesophageal cancers: OR = 1.20; 95% CI 0.10–14.40	3A	Poor
Wyss [48]	6772 cases, 8375 controls	oral, pharyngeal, and laryngeal cancers	Head and neck cancer (HNC): OR = 1.71; 95% CI 1.08–2.70	3A	Poor
Cardiovascular					
Gupta [9]	20 studies	CHD	OR = 1.04; 95% CI 0.83–1.24	2A	Good
Obisesan [47]	13,086	Hypertension	Odds of hypertension: 0.88 times lower (95% CI 0.7907–0.9896)	2C	Fair
Gupta [10]	14 studies	IHD Mortality, stroke	Stroke: OR = 1.21; 95% CI 0.90–1.51 Fatal stroke: OR = 1.21; 95% CI 0.90–1.51	3A	Poor
Rostron [46]	24 studies	IHD, stroke	IHD: RR = 1.17; 95% CI 1.08–1.27 Stroke: RR = 1.28; 95% CI 1.01–1.62	3A	Good
Other					
Hernandez [41]	4930	Self-reported chronic health conditions	At least one chronic health condition: OR = 1.49; 95% CI 1.23–1.81	2C	Poor
King [49]	2370	Self-reported depression, stress, mental health diagnosis	Mental health diagnosis: AOR = 1.16; 95% CI 0.64–2.10; p = 0.594 Depression: AOR = 0.98; 95% CI 0.95–1.00 Stress: AOR = 1.03; 95% CI 1.00–1.05	2C	Fair
*Europe*					
Mortality					
Araghi [50]	417,872	All-cause mortality, colorectal cancer	All-cause mortality: HR = 1.16; 95% CI 0.89, 1.50	2A	Fair
Wilson [51]	9582	Prostate cancer mortality, overall mortality	Overall mortality: HR = 1.19; 95% CI 1.04–1.37 Prostate cancer-specific mortality: HR = 1.24; 95% CI 1.03–1.49	2B	Fair
Gupta [10]	of 14 studies	IHD Mortality, stroke	Fatal stroke: OR = 1.30; 95% CI 0.96–1.63	3A	Poor
Sinha [12]	16 studies	Mortality, all cancer, UADT cancer, stomach cancer, cervical cancer, IHD, stroke	IHD mortality: OR = 1.16; 95% CI 1.05–1.28	3A	Poor
Vidyasagaran [11]	19 studies	IHD	IHD death: OR = 1.38; 95% CI 1.13–1.67 Stroke death: OR = 1.28; 95% CI 0.98–1.68	3A	Good
*Morbidity*					
Cancer					
Araghi [50]	417,872	Colorectal cancer, all–cause mortality, cancer-specific mortality	Colorectal cancer: HR = 1.22; 95% CI 0.91, 1.64 Colon cancer: HR = 1.02; 95% CI 0.81–1.29 Rectal cancer: HR = 1.29; 95% CI 1.02–1.30	2A	Fair
Araghi [53]	418,488	Pancreatic cancer	HR = 0.96; 95% CI 0.83–1.11	2A	Fair
Asthana [52]	37 studies	Oral cancer	OR = 0.86; 95% CI 0.58–1.29	3A	Poor
Siddiqi [13]	32	Oral, pharyngeal, oesophageal cancers	Mouth (oral cavity, tongue, and lip) cancers: Sweden: OR = 0.92; 95% CI 0.68–1.25; Norway: OR = 1.10; 95% CI 0.5–2.42 Pharynx cancer: Sweden: OR = 1.45; 95% CI 0.3–6.21 Oesophageal cancers: Norway: OR = 1.40; 95% CI 1.61–3.21; Sweden: OR = 1.26; 95% CI 1.02–1.56	3A	Poor
Cardiovascular					
Gupta [9]	20 studies	CHD	OR = 0.95; 95% CI 0.86–1.04	2A	Good
Gupta [10]	14 studies	IHD Mortality, stroke	Stroke: OR = 1.04; 95% CI 0.94–1.15 Fatal stroke: OR = 1.30; 95% CI CHD-1.63	3A	Poor
Rostron [46]	24 studies	IHD, stroke	IHD: RR = 1.04; 95% CI 0.93–1.16 Stroke: RR = 1.04; 95% CI 0.92–1.17	3A	Good
Vidyasagaran [11]	19 studies	IHD	IHD: OR = 0.91; 95% CI 0.83–1.01 Stroke: OR = 1.01; 95% CI .90–1.13	3A	Good
Other					
Rauwolf [83]	347	Alcohol dependence	No significant differences between the four groups regarding total abstinence (i.e. no alcohol consumption after end of treatment), days of alcohol consumption the last 30 days or grams of pure alcohol per week	2B	Fair
Yang [54]	348,601	Parkinson’s	60% lower Parkinson’s disease risk/HR = 0.41; 95% CI 0.28–0.61	2A	Good
Gudnadóttir [55]	16,082	Asthma, respiratory symptoms, sleep-related problems	Asthma: OR = 1.49; 95% CI 1.20–1.85 Chronic bronchitis: OR = 1.47; 95% CI 1.21–1.78 Chronic rhinosinusitis: OR = 1.37; 95% CI 1.11–1.70	2C	Good

*Level of Evidence assigned using the Oxford Centre for Evidence Based Medicine framework [8]

### Health outcomes by region

#### Global

Six studies reported on combined global SLT-product data [10–14, 53].

### Mortality

A “good”-rated meta-analysis of 20 studies on snuff (not Swedish snus), chewing tobacco and naswar from Europe, the USA, Southeast Asia and the Mediterranean region found a borderline association of combined SLT-products and fatal CHD (OR = 1.10; 95% CI 1.00–1.20), higher risk with naswar (OR = 1.30; 95% CI 1.06–1.54) but not chewing tobacco, in smoking-adjusted studies [10]. A “poor”-rated meta-analysis of 14 studies in Europe, USA, Southeast Asia and Mediterranean found combined SLT-product users had higher risk of fatal stroke (OR = 1.27; 95% CI 1.15–1.39) after excluding or adjusting for smoking [11]. Another “good”-rated meta-analysis of 19 studies from North America, Asia, and Europe found increased risk of deaths from ischaemic heart disease (IHD) (OR = 1.15; 95% CI 1.01–1.30) and stroke (OR = 1.39; 95% CI 1.29–1.49) in SLT-product ever-users compared with never tobacco-users [12].

A “poor”-rated meta-analysis that pooled together different SLT-products from 16 global studies reported increased risk of overall mortality (OR = 1.22; 95%I: 1.11–134), with significant heterogeneity [13].

A “poor”-rated meta-analysis of 32 global studies estimated 1.7 million disability-adjusted life years (DALYs) lost and 62,283 deaths in 2010 globally from cancers of the mouth, pharynx and oesophagus attributed to SLT-product [14]. Most included studies adjusted for but didn’t exclude smoking.

### Cardiovascular outcomes

One 'good'-rated meta-analyses of 19 studies on chewing tobacco, dip, snuff and snus from Sweden, North America, and Asia found no increase in IHD for combined regions (OR = 1.4; 95% CI 0.92–1.42) in studies that excluded former smokers [12].

Another ‘good’-rated meta-analysis of 20 studies from four WHO regions including snuff, chewing tobacco and naswar found no increased risk of CHD overall (OR = 1.05; 95%CI 0.95–1.16) or for chewing tobacco (OR = 1.13; 95% CI 0.92–1.06), but did for naswar (OR = 1.30; 95% CI 1.06–1.54), including studies that excluded or adjusted for smoking [10].

A ‘poor’-rated global meta-analysis on SLT-products, which did not account for variation in handling of smoking status, reported an association with stroke overall (OR = 1.18; 95% CI 1.04–1.32) and chewing tobacco (OR = 1.35; 95% CI 1.20–1.50) but not for snuff (OR = 1.03; 95% CI 0.93–1.13) or naswar (OR = 0.98; 95% CI 0.57–1.39) [11].

### Cancer

#### Oral Cancer

A ‘poor’-rated global meta-analysis of 32 studies found an association with oral cancer overall (OR = 3.43; 95% CI 2.26–5.19) [14].

#### Head and Neck Cancer (HNC)

A ‘poor’-rated meta-analysis reported an association with pharyngeal cancer for all countries combined (OR = 2.23; 95% CI 1.55–3.20) and India (OR = 2.60; 95% CI 1.76–3.85); and oesophageal cancer for all countries combined (OR = 2.17; 95% CI 1.70–2.78), India (OR = 2.57; 95% CI 2.20–3.00) and Pakistan (OR = 8.20; 95% CI 1.45–27.47) [14].

#### Other cancers

A meta-analysis of 16 global studies found combined SLT products were associated with mortality due to cancers overall (HR = 1.31; 95% CI 1.16–1.47), of upper aero digestive tract (UADT) (HR = 2.17; 95% CI 1.47–3.22), stomach (HR = 1.33; 95% CI 1.12–1.59 and cervix (HR = 2.07; 95% CI 1.64–2.61) [13].

#### SE Asia, Middle East, Africa (AMEA)

### 42 studies reported on SLT-product data from AMEA [10–40, 53, 80–84], 32 exclusively from AMEA region [15–40, 80–84].

### Mortality

A ‘poor’-rated meta-analysis of 16 global studies reported increased overall mortality for South East Asian (SEAR) (OR = 1.25; 95% CI 1.08–1.44), with significant heterogeneity [13]. A longitudinal study of 50,045 participants from Iran found naswar use in never-smokers was associated with increased overall mortality (HR = 1.17; 95% CI 1.00–1.36) and cancer mortality (HR = 1.40; 95% CI 1.01–1.95), which was further elevated in dual (cigarette and naswar) users (overall mortality: HR = 1.28; 95% CI 1.00–1.64; cancer mortality: HR = 1.67; 95% CI 1.02–2.75); there was no elevated risk of IHD, CVD or respiratory mortality [15]. A ‘poor’-rated Indian case–control study found increased overall (RR = 1.3; 95% CI 1.2–1.4) and respiratory mortality (RR = 1.5; 95% CI 1.4–1.7) for chewing tobacco users amongst never-smokers [16].

### Cardiovascular outcomes

One good quality global meta-analyses on 19 studies of SLT-products (chewing tobacco, dip, snuff and snus) found increased IHD risk in Asia (OR = 1.40; 95%CI 1.01–1.95) after excluding former smokers [12]. A poor-rated meta-analysis reported an association between SLT-products and stroke in SE Asia (OR = 1.35; 95% CI 1.18–1.51), but not in Mediterranean [11]. A poor-rated cohort study in India reported increased stroke (OR = 3.71; 95% CI 1.57–9.05) and myocardial infarction (MI) (OR = 2.34; 95% CI 1.10–5.40) in SLT users without excluding former smokers [17].

An Indian cross-sectional study reported no increased diabetes or hypertension in exclusive SLT-product users [18], although former smoking was not accounted for. An Indian cross-sectional study of 36 individuals with mental and behavioral disorders reported no association with CHD compared with exclusive smokers [19]; another study with 30 exclusive SLT-product users reported strong associations with dyslipidaemia (OR = 6.37; 95% CI 1.4–27.3) and hypertension (OR = 6.97; 95% CI 1.7–28.0) compared with non-tobacco users [20].

### Cancer

Of 25 studies on cancer in AMEA region [14, 21–29, 31–40, 53, 80–82], only one was rated as ‘good’ [21].

#### Oral cancer

A ‘good’-rated meta-analysis in Pakistan reported an association between naswar use and oral cancer (OR = 11.8; 95% CI 11.4–25.3), four of six studies had adjusted for smoking [21]. A ‘poor’-rated meta-analysis of 32 global studies reported an association between combined SLT-products and oral cancer in India (OR = 5.12; 95% CI 3.27–8.02) and Pakistan (OR = 8.81; 95% CI 3.14–24.69) [14].

A ‘poor’-rated Indian meta-analysis of 25 studies reported increased oral cancer risk with combined SLT-products (OR = 5.65; 95% CI 3.83–8.40) [22]. A ‘poor’-rated meta-analysis of Shammah use in Middle East and North Africa [23], in which only one of three studies adjusted for smoking, reported elevated risk of oral cancer (OR = 38.7; 95% CI 19.50–76.96).

Of several small, predominantly hospital-based, case–control studies, two in Saudi Arabia found elevated oral cancer with exclusive Shammah use (OR = 29.30; 95% CI 10.33–83.13) [24], (OR = 33.01; 95% CI 3.22–39.88) [25] and lower risk with dual use of shammah and cigarettes (OR = 10.10; 95%CI = 0.50–20.40) [24]. A study in Pakistan found elevated risk with gutka (OR = 5.54; 95% CI 2.83–10.83) and chewing tobacco (OR = 5.32; 95% CI 1.14–24.77) [26]. An Indian study reported elevated risks with chewing tobacco (OR = 8.51; 95% CI 4.90–14.77) [27], and a Sudanese study from Tokomak dipping (OR = 3.8; 95% CI 1.7–8.6), after adjusting for smoking [28]. A study of naswar use in Pakistan reported elevated risk for current users (OR = 23.4; 95% CI 6.6–82.1), ever users (OR = 21.0; 95% CI 6.1–72.1) and former users (OR = 16.4; 95% CI 4.1–65.4), after adjusting for smoking [29]. An Indian study reported elevated risk of oral cancer for combined SLT-products (OR = 6.0; 95% CI 2.6–15.5), gutkha (OR = 5.1; 95% CI 2.0–10.3), supari (OR = 11.4; 95% CI 3.4–38.2) and betel quid (OR = 6.4; 95% CI 2.6–15.5), but not for snuff (OR = 1.0; 95% CI 0.3–3.0), after adjusting for smoking [30]. Another Indian study reported elevated risk in sole chewing tobacco users (OR = 2.8; 95% CI 1.2–7.0) but not in dual users (OR = 0.7; 95% CI 0.2–2.6) [31]. A case–control study in Pakistan reported elevated risk for combined SLT-products (OR = 4.71; 95% CI 2.53–8.74), snuff (OR = 4.82; 95% CI 2.37–9.80), betel leaf (OR = 4.42; 95% CI 1.66–11.91) and supari/chalia (OR = 4.67; 95% CI 1.14–19.12) after adjusting for smoking [32]. We found in addition two case series [33, 34] and one ecological study [35].

#### Head and neck cancer

Of studies investigating SLT-product use and head and neck cancer (HNC), a ‘poor’-rated meta-analysis reported an association with pharyngeal cancer in India (OR = 2.60; 95%CI = 1.76–3.85) and oesophageal cancer in India (OR = 2.57; 95% CI 2.20–3.00) and Pakistan (OR = 8.20; 95% CI 1.45–27.47) [14]. A ‘poor’-rated meta-analysis from India reported an association between combined SLT-products and pharyngeal (OR = 2.69; 95% CI 2.28–3.17) and oesophageal cancers (OR = 3.17; 95% CI 2.76–3.63) [22].

A case–control study from Nepal reported an association between chewing tobacco and HNC (OR = 2.39; 95% CI 1.77–3.23), higher with heavy use (≥ 6 times per day) (OR = 2.91; 95% CI 2.06–4.12) and duration over 20 years (OR = 2.92; 95% CI 2.08–4.11) [36]. One study described the commonest sites for chewing tobacco related HNC cancer as the gingivobuccal complex [33]. An ecological analysis of regional population-based cancer registries in India found correlations for Khaini use and hypopharynx cancer (r = 0.48 males, r = 0.29 females), gutka use and mouth cancer in males (r = 0.54, r = − 0.19 for females) and oral tobacco and mouth cancer in males and females (r = 0.46 males, r = 0.17 females) [35] ‘Other’ types of SLT-product use (combined) correlated with hypopharynx cancer (r = 0.47). The study did not account for smoking.

#### Other cancers

Two hospital-based case–control studies reported associations between chewing tobacco and breast cancer (OR = 2.35; 95% CI 1.3–4.15) [37] (OR = 2.35; 95% CI 1.01–5.51) higher in heavy users (> 5 times daily)(OR = 10.13; 95% CI 5.41–18.23) and duration ≥ 10 years (OR = 31.13; 95% CI 11.67–39.82) [38]. A ‘poor’-rated Indian meta-analysis reported associations between combined SLT-products and stomach (borderline significance, OR = 1.26; 95% CI 1.00–1.60) and laryngeal cancers (OR = 2.84; 95% CI 2.18–3.70); both were non-significant in random effects models (OR = 1.31; 95% CI 0.92, 1.87, OR = 1.79; 95% CI 0.70–4.54) and there was no association with lung cancer (OR = 0.91; 95% CI 0.76–1.09) [22]. A hospital-based case–control study in Yemen found SLT-product use to be associated with gastric cancer (OR = 4.37; 95% CI 1.92 to 9.95), but not with cigarette smoking [39]. An Indian hospital case–control study found SLT-product use to be associated with colorectal cancer (OR = 1.53; 95% CI 0.58–4.00) after adjusting for cigarette smoking [40].

### Other health outcomes

A hospital case–control study of Indian chewing tobacco users reported greater gingival bleeding (OR = 1.710; 95% CI 1.2–2.43), loss of attachment (OR = 2.393; 95% CI 1.55–3.69) and attrition (OR = 2.496; 95% CI 1.73–3.61) [41]. Other Indian studies reported self-reported chronic health conditions [42], obstetric and neonatal health but not of gastro-intestinal, urinary disease [43] or asthma [18], and reduced chronic lung disease (OR = 0.64; 95% CI 0.45–0.91) in SLT-product users [18].

#### USA

Of 15 studies reporting on SLT-product data from USA [10–14, 42–50, 53], eight were exclusively in USA [42–46, 48–50, 53].

### Mortality

A large US study constituting a high level of evidence pooling two longitudinal studies found no increase in mortality overall or due to smoking-related cancers or CVD in never smoking SLT-product users compared with never-smoking never-SLT-product users [43]. Dual users of SLT-product and cigarettes had similar excess mortality (HR = 2.21; 95% CI 1.50–3.26-HR = 2.14; 95% CI 1.27–3.59) to exclusive smokers (non-SLT-product users) (HR = 2.10; 95% CI 1.99–2.22-HR = 1.88; 95% CI 1.75–2.02), compared with never tobacco users.

A large ‘good’-rated US longitudinal study found exclusive SLT-product users had increased all-cause mortality (HR = 1.44; 95% CI 1.12–1.84) but not cause-specific mortality [44]. A large ‘fair’-rated US longitudinal study that excluded former and current smokers, but included both reported on snuff and chewing tobacco together, reported higher CHD mortality (OR = 1.25; 95% CI 1.05–1.46) but not mortality overall or from cancer and other types of CVD [45]. A large and ‘good’-rated US population-based cohort study reported higher overall mortality (HR = 1.36; 95% CI 1.17–1.59), CHD mortality (HR = 1.63; 95% CI 1.27–2.09), cancer mortality (HR = 1.48; 95% CI 1.04–2.12) and smoking-related cancer (HR = 1.76; 95% CI 1.07–2.90), but not respiratory-related or CVD mortality in SLT-using never-smokers [46]. Higher risk of overall mortality was only seen with daily SLT use (HR = 1.41; 95% CI 1.20–1.66) and not with less than once daily use.

A ‘poor’-rated meta-analysis of 16 studies globally reported from US data an increased risk of mortality overall (OR = 1.17; 95% CI 1.12–1.22) and due to cancer (OR = 1.14; 95% CI 1.01–1.29), stroke (OR = 1.44; 95% CI 1.30–1.59), and IHD (OR = 1.16; 95% CI 1.05–1.28); there was significant heterogeneity but no publication bias [13].

### Cardiovascular outcomes

A ‘good’-rated meta-analysis of SLT-products (including snuff and chewing tobacco) of 24 US studies reported elevated IHD risk (RR = 1.17; 95% CI 1.08–1.27) and stroke (RR = 1.28; 95% CI 1.01–1.62) compared with non-users, despite variation in handling of smoking status [47]. A ‘poor’-rated meta-analysis reported no association between SLT-products and stroke in US data [11]. A US cross-sectional study reported lower self-reported hypertension (OR = 0.88; 95% CI 0.79–0.98) in SLT-product users (adjusted for smoking status and duration) [48].

### Cancer

#### Oral cancer

A ‘poor’-rated review that pooled 11 US studies found SLT-products (snuff and chewing tobacco) to be associated with cancers of oral cavity (OR = 1.81; 95% CI 1.04, 3.17) [49]. A large ‘poor’-rated global meta-analysis found no association with oral cancer in North American data [14].

#### Head and neck cancer

The largest study investigating HNC, a ‘poor’-rated global MA [14], reported for pharyngeal and oesophageal cancers, respectively: associations for all countries combined (OR = 2.23; 95% CI 1.55–3.20; OR = 2.17; 95% CI 1.70–2.78) but not for North America (single study only). A review that pooled 11 US studies found increased odds for HNC in snuff users (OR = 1.71; 95% CI 1.08–2.70) but not for ever-tobacco chewers, compared with never users [49]. with a dose–response effect with increasing duration of snuff use (p-value for trend = 0.007).

#### Other cancers

There were no exclusive US data on other cancers.

### Other health outcomes

One US cross-sectional study reported no significant association between SLT-product use and a diagnosis of mental health disease or depression [50].

#### Europe

Of 16 studies reporting on SLT-product data from EU [10–14, 47, 51–56, 85], seven were exclusively from EU [51–56, 85].

## Mortality

A large ‘fair’-rated study pooling nine Swedish cohort studies found no association between exclusive current snus use and all-cause mortality (HR = 1.16; 95% CI 0.89–1.50), compared with never-smoking non-snus users [51]. A ‘fair’-rated cohort study on Swedish prostate cancer patients reported increased overall mortality (HR = 1.19; 95% CI 1.04–1.37) in snus users compared with non-snus users, in never smokers, and a similar risk for dual snus and cigarette (OR = 1.17; 95% CI 1.06–1.28) [52]. A ‘poor’-rated global meta-analysis of 16 studies reported in EU data no increased all-cause, cancer or stroke mortality, but elevated risk of IHD mortality (OR = 1.16; 95% CI 1.05–1.28) [13].

### Cardiovascular outcomes

Four meta-analyses, three global, including 14 [11], 20 [10] and 19 global [12] studies, and one of 24 EU studies [47], of which three were rated as ‘good’ [10, 12, 47], and all of mixed study-designs, found no association between snus use and IHD (RR = 1.04; 95% CI 0.93–1.16) [47], (OR = 0.91; 95% CI 0.83–1.01) [12], CHD (OR = 0.93; 95% CI 0.81–1.06) [17], or stroke (OR = 1.04, 95% CI 0.94–1.15) [11], (RR = 1.04; 95% CI 0.92–1.17) [47], (OR = 1.01; 95% CI 0.90–1.13) [19] in studies that excluded former smokers.

### Cancer

#### Oral cancer

There were no region-specific studies of oral cancer in Europe. A ‘poor’-rated global meta-analysis showed no association between combined SLT-products oral cancer in Sweden or Norway [14]. A ‘poor’-rated meta-analysis on 37 global case–control and cohort studies found no association between snus and moist snuff use and oral cancer in European data [53].

#### Head and neck cancer

A ‘poor’-rated meta-analysis of combined SLT-product use showed no association with pharyngeal cancer but excess risk of oesophageal cancer (OR = 1.26; 95% CI 1.02–1.56) in Sweden and in a single study from Norway (OR = 1.40; 95% CI 0.61–3.21) [14].

#### Other cancer

A large ‘fair’-rated review of nine Swedish cohort studiesfound no association with colorectal cancer for current (HR = 1.22; 95% CI 0.91, 1.64) or former exclusive snus users (HR = 1.12; 95% CI 0.75, 1.67); no association with colon cancer (HR = 1.02; 95% CI 0.81, 1.29) in current exclusive snus users but increased risk of rectal cancer in current snus users (HR = 1.38; 95% CI 1.07, 1.77) in never-smokers, with no dose–response effect for quantity or duration [51]. No association was found with pancreatic cancer pooling the same Swedish cohort studies [54].

### Other health outcomes

A large, ‘good’-rated meta-analysis of Swedish cohort studies reported considerably lower Parkinson’s disease risk in never-smoking snus users (pooled HR = 0.41; 95% CI 0.28–0.61), with lower risk for moderate-heavy snus quantity (pooled HR = 0.41; 95% CI 0.19–0.90) and long-term duration (pooled HR = 0.44; 95% CI 0.24–0.83) [55]. Moderate-heavy snus quantity (pooled HR = 0.41; 95% CI 0.19–0.90) and long-term current-snus use (pooled HR = 0.44; 95% CI 0.24–0.83) had lower risk. One Swedish cross-sectional study reported increased asthma (OR = 1.49; 95% CI 1.20–1.85), chronic bronchitis (OR = 1.47; 95% CI 1.21–1.78) and chronic rhinosinusitis (OR = 1.37; 95% CI 1.11–1.70) in snus-using never smokers [56].

## Discussion

This is one of the first articles to systematically review health outcomes from SLT product use, and in particular, to differentiate between the different types of products used in Asia, Middle East and Africa, Sweden, other parts of Europe and the US.

Most studies were from AMEA and were less likely to be of rigorous study design than those from Europe and the USA. Two-thirds of global studies and a half of US studies evaluated mortality (66%; 50%), whereas AMEA studies mostly evaluated cancer (23; 72%). Meta-analyses made up 100% of global studies and 57% of Europe studies. Case–control represented 50% of AMEA studies.

Methodological flaws with the greatest impact included combining different SLT-products as seen in the global meta-analyses [10–14, 53], and widespread failure to adequately account for dual and former cigarette smoking.

### Health outcomes

Results indicate stark differences for health outcomes for different SLT-products and regions. There is overwhelming evidence that SLT-products in AMEA are associated with harmful health outcomes, including higher mortality: strongly for overall, cancer, CHD; less so for respiratory mortality and not shown to increase overall CVD mortality; increased CVD morbidity, with strong associations for IHD and stroke, and mixed evidence for hypertension and dyslipidaemia.

Different SLT-products, even within the same region, have varied strengths of association with oral cancer, with odds ratios ranging from 29 to 39 for shammah; 23 for naswar, 11 for supari, 5.5 gutkha, 8.5 for chewing tobacco and 3.8 for tokomak dipping compared to non-use. All types of SLT-products used in AMEA were associated with head and neck cancers albeit with lower odds than for oral cancer, of up to 3.2.

In stark contrast, the fewer but higher-quality studies in Europe, predominantly in Sweden, found snus and other SLT-products not to cause higher mortality or morbidity overall or from overall mortality, CVD or cancers. Two high quality meta-analyses showed no excess mortality, although one smaller cohort study contradicted this finding. Five meta-analyses found no excess IHD risk, and four found no excess stroke risk. There was no excess oral or head and neck cancers, pancreatic or colon cancer, but raised risk of rectal cancer in one study [51] and harms to respiratory disease from snus use [56]. There was robust evidence from pooled studies for a protective effect of snus against the development of Parkinson’s disease (by more than 50%) [55]. The differences in detrimental health outcomes seen between snus users in Sweden and other parts of Europe compared to elsewhere may in part be attributable to the different chemical content [57].

US studies showed more mixed results from SLT-product use with some evidence of harmful health outcomes. Meta-analyses and longitudinal studies showed mixed results for overall mortality, and mortality due to CHD, overall cancer and smoking-related cancers but no excess risk of respiratory or CVD mortality. Risk of non-fatal CVD were also mixed but the most rigorous study reported elevated risk for both IHD and stroke [47]. A single cross-sectional study reported reduced hypertension rates in SLT-product users. There were mixed results for oral and head and neck cancers ranging from no excess risk to a pooled odds ratio of 1.8 [49].

No studies of more novel products such as tobacco-free nicotine pouches were captured. Of the 53 studies, none reported on the health impact of switching from cigarettes to SLT-products.

### Levels of evidence, quality and study design

No studies were above 2a for level of evidence [8]. There were no meta-analyses, pooled studies, or indeed individual interventional studies, which perhaps reflects difficulty conducting these in real world settings. Meta-analyses comprised the most common study design (21 studies); despite being large, including over 30 studies [14, 53] and 350,000 participants [54, 55], only five of the 21 meta-analyses rated as ‘good’ [10, 12, 21, 47, 55]. Particularly problematic themes included pooling different SLT-products, failing to account for heterogeneity of studies, pooling studies despite variation in sampling methodologies, and failing to report country-specific results, even when these were available.

Case–control and cross-sectional studies also predominated, both which are problematic in terms of accounting for bias, such as failing to account for temporality of exposure and outcome, as well as former smoking status, rendering cross-sectional studies inappropriate for causal inferences. Two-thirds of global and half of AMEA region studies were rated as being of ‘poor’ quality; all studies exclusively from Europe and two-thirds of those from USA were rated as ‘good’ or ‘fair’.

#### Definitions of exposures

Studies frequently failed to account for quantity and duration of SLT-product use, dual and former use of cigarettes, and in former smokers, duration since quitting. Standard definitions exist for smoking that consider both quantity and duration [58] and similar approaches should be used for SLT-products. Furthermore, a strong dose response effect has been demonstrated in several studies for both quantity and duration of SLT-product use in AMEA, which should form part of the measurement of exposure.

#### Accounting for smoking status

Indian SLT-product users often smoke concurrently [59, 60] and it is essential for both dual and former cigarette use to be accounted for when investigating health outcomes. Of snus use in Sweden, 82% were former or dual users of cigarettes [61]. In our review, only nine studies accounted for both former and current smoking, four out of 11 studies in USA and Europe, and six out of 49 studies from global and AMEA regions.

#### Publication bias

No formal evidence for publication bias was found in many of the meta-analyses in our review. However, the small number of studies investigating SLT-products in Sweden, Europe and US suggests that this is an under-researched area and the preponderance of reporting on negative outcomes could indicate the presence of publication bias.

#### Role of SLT-products in reducing smoking rates

SLT-product use in India represents two-thirds of all global SLT use [62] with prevalence rates of 30% in men and 13% in women, exceeding those for cigarettes (7% men, 0.6% women) and bidis (14% men, 1.2% women).

The use of snus by smokers has been associated with decreased cigarette smoking and increased abstinence of smoking [63–69]. Other studies do not support some of these findings [68, 70, 71]. Some have postulated snus use in Sweden has led to low smoking prevalence rates through a “reverse gateway” effect [69]. The low prevalence of smoking in favour of snus use in Sweden compared to the rest of Europe may have contributed to its lower rates of tobacco-attributable deaths (72/100,000 Sweden, 128/100,000 EU) and cancer-specific deaths (14/100,000 Sweden, 36/100,000 EU) in men in 2019 [72]. This strengthens the argument for safer forms of SLT-products such as Swedish snus to be used as a form of tobacco harm reduction on the pathway to stopping smoking. Indeed, data from Swedish longitudinal studies show in primary smokers who started secondary snus use, 10.6% reduced to occasional smoking and 76.3% stopped smoking altogether [5]. Furthermore, between 40 and 50% of secondary snus users later also quit snus use (during 7 years of follow up) [5, 74], Modelling has suggested switching from smoking to Swedish snus is likely to result in net health gains [74].

### Informing Policy

The findings of our review have implications for policy makers. SLT-products are subject to regulations with regard to sales restrictions, advertising, packaging and labelling.[75] Sweden has demonstrated that through strong regulation of composition, SLT-product-related harm has been minimised [76]. The Tobacco Products Directive (TPD) in the European Union has issued a total ban on Swedish snus outside of Sweden whilst allowing South-East Asian SLT-products [77], a policy which is contradicted the findings of our review and previous scientific evidence. The findings of this review, together with growing evidence of their role in reducing smoking rates, do not support the continuation of a ban on Swedish snus and other tobacco harm reduction products as a safer alternative to cigarette smoking.

### Strengths and limitations

It’s a challenge to estimate the risk of disease attributable to such a heterogeneous risk factor such as SLT-products [13]. Any review involving SLT-products will be limited by these issues, unless a single product is studied such as the European snus or the Asian naswar [13]. The output of our systematic review is thus limited due to its reliance on studies which have reported on heterogenous SLT-products. Furthemore, a meta-analysis of included studies could not be undertaken due to the methodological flaws and vast heterogeneity between studies.

We summarized findings by region and reported on different products as the best ‘fit’ for categorization of SLT-product use. However, this is not perfect due to the changing landscape and product variation within regions. This issue will only be resolved by future studies carefully documenting and reporting separately for each type of SLT-product.

We sought to identify only those articles where the main research question was on health outcomes from use of SLT-products. The key health outcomes under investigation were mortality, CVD, respiratory and cancer as these make up the major health concerns from SLT-products. We also searched for general health outcomes to identify the breadth of health outcomes being reported.

Finally, the search strategy results were limited to English language reports, and there is a risk that potentially relevant studies reporting health outcomes with ENDS use were subsequently not included.

## Conclusion

Our review found studies on SLT-product use focus predominantly on negative health impacts and no studies were found on the health impact from switching from cigarettes to SLT-products. The strength of evidence and quality of the published studies are generally poor, particularly for global studies and those from Asia, Middle East and Africa.

Our review found large differences on the impact on health outcomes between different SLT-products in different regions. Use of SLT-products in Asia, Middle East and Africa region is associated with harmful health outcomes including higher overall and cancer mortality, CVD morbidity, and greatly increased morbidity from most smoking-related cancers, in particular oral cancer. In stark contrast, SLT-products used in Sweden and other parts of Europe such as snus have not been shown on the whole to cause higher mortality or morbidity from CVD or most cancers with evidence for a protective effect against the development of Parkinson’s disease. SLT-product use in the US shows more mixed results for mortality, CVD and cancer outcomes with a higher risk than for Europe but substantially lower than those from SE Asia, Middle East and Africa.

Further studies are required to investigate health outcomes from switching from cigarettes to SLT-products and to investigate the full breadth of health outcomes. The wider impacts from SLT-product use on society, such as new uptake in never smokers and nicotine addiction as must also be considered.

Considering the widespread and increasing use of SLT-products in certain parts of the world, there is far less evidence base for their impact on health outcomes compared with cigarette smoking, which is in part due to their predominant use in developing countries. However, the emergence of SLT-products as a driver for reduced smoking rates in Sweden and other parts of Europe warrant further clarification of risk from specific and novel SLT-products.

### Supplementary Information

**Additional file 1: Table 5**.

## Appendix 1

There are numerous types of SLT-products available globally which differ markedly in terms of their preparation, method of use and toxicity [6].

Indian SLT-products undergo fermentation which affects the production of potential carcinogens called tobacco-specific nitrosamines (TSNAs) [84, 85] and are often combined with additives such as betel leaf (*Piper betle*), sliced areca nut (*Areca catechu*) and/or powdered agricultural lime [86], which further enhances their toxicity and psychotropic effect [87, 88].

Even in western countries, SLT-products are not a homogeneous category [89]. In the US, three traditional types of SLT-products are used: powdered dry snuff, loose leaf chewing tobacco and moist snuff although use of the former two has rapidly declined [90]. In Scandinavia, especially in Sweden, there is a long tradition of moist snuff use, where 'snus' (the generic term for moist snuff in Swedish, pronounced 'snoose') is essentially the only type of SLT-product in use [91].

In the US, dry snuff is made from fermented, fire-cured tobacco that is pulverized into powder. Loose-leaf chewing tobacco consists of air-cured leaf tobacco. Moist snuff usually consists of fire-cured dark tobaccos and is used by a 'pinch' between the thumb and forefinger and placing inside the lip [90].

The fermentation of traditional American products results in higher concentrations of unwanted bacterially mediated by-products, especially TSNAs and nitrite. In Swedish manufacturing of snus air cured tobacco leaves are subjected to pasteurization, yielding virtually sterile products containing very low levels of TSNAs. The manufacturing of Swdish snus does not involve fermentation. Instead, the air cured tobacco leaves are subjected to a heating process (pasteurization), yielding virtually sterile products containing very low levels of TSNAs. Further, in Sweden’s manufacturing of snus the tobacco leaves are processed according to Swedish legal regulations for food products and the rigorous, industry standard “GothiaTek”. Therefore, physical and chemical characteristics of US and Swedish snus products can vary considerably and should not be considered “equivalent” [57].

Other SLT-products also exist, for example, traditional tobacco pouches may contain moist or dry snuff, or small pieces of leaf tobacco and pellets of compressed tobacco. Nicotine pouches contain either tobacco-derived nicotine or synthetic nicotine, but no tobacco leaf, dust, or stem, and are described as either similar to or being a tobacco-free version of snus.

The commonest SLT-products globally are shown in Table 1 (Copied from: IARC Monographs on the Evaluation of Carcinogenic Risks to Humans [6].

## Appendix 2

### Search terms

Search terms for SLT: Smokeless tobacco; SLT, Chewing tobacco; Reduced risk tobacco; Non-cigarette tobacco; Snus; Snuff

Search terms for health outcomes: Health outcome; Morbidity; Mortality; Cancer; Cardiovascular disease; Chronic obstruct pulmonary disease; COPD; CVD; Acute myocardial infarction; Stroke; Cardiovascular; Cerebrovascular; Health effects; Adverse; effects; Respiratory.

## Abbreviations

AMEA: Asia, Eastern Mediterranean and Africa
AMR: Americas Region
BMI: Body mass index
CAD: Coronary artery disease
CHD: Coronary heart disease
COPD: Chronic obstructive pulmonary disease
CVD: Cardiovascular disease
DALYs: Disability-adjusted life years
DBP: Diastolic blood pressure
EUR: European Region
HNC: Head and neck cancer
IHD: Ischemic heart disease
MI: Myocardial infarction
SBP: Systolic blood pressure
SEAR: Southeast Asian Region
SLT: Smokeless tobacco
