# Supplementary material for: The health impact of smokeless tobacco products: a systematic review
Source: Harm Reduct J. 2021 Dec 4;18:123. doi: 10.1186/s12954-021-00557-6 (PMC8643012; doi:10.1186/s12954-021-00557-6)
Supplement: Supplementary file 1 — Additional file 1: Table 5. [file 12954_2021_557_MOESM1_ESM.docx]

**Table 5**. Description of Studies, Level of Evidence and Quality for Health Outcomes from SLT by Region.

| **Reference** | | **Study Design & Number of Participants** | **Country** | **Key Outcomes assessed** | **Impact on Health Outcome** | **Level of Evidence** | **Quality Rating** |
| --- | --- | --- | --- | --- | --- | --- | --- |
| **GLOBAL** | | | | | | | |
| **MORTALITY** | | | | | | | |
| Gupta, et al. Association of smokeless tobacco and cerebrovascular accident: a systematic review and meta-analysis of global data | | Meta-analysis of 14 studies | Global (EU, SEA, Americas, EMR) | IHD Mortality, stroke | Among smoking adjusted studies (excluded smokers or adjusted for), and across all regions (Europe, US, Southeast Asia, Mediterranean), compared to non-users of SLT, exclusive SLT users (all types) had higher risk of fatal stroke (OR=1.27; 95% CI: 1.15-1.39). No significant publication bias was found in the included studies. Only those studies were included in the analysis that had exclusive SLT users as subjects or which included smokers as well as SLT users but listed separate results for SLT users. Heterogeneity was limited in all regions except the US studies (66%). | **3A** | **Poor** |
| Siddiqi, et al. Global burden of disease due to smokeless tobacco consumption in adults: analysis of data from 113 countries. | | Meta-analysis and comparative risk assessment of 32 cohort and case-control studies | Global (South East Asia, Europe, North America) | DALYs and mortality | In 2010, SLT use led to 1.7 million, DALYs lost and 62,283 deaths due to cancers of mouth, pharynx and oesophagus. No tests for publication bias. Most studies adjusted for, but did not exclude, smoking. | **3A** | **Poor** |
| Sinha, et al. Global burden of all-cause and cause-specific mortality due to smokeless tobacco use: systematic review and meta-analysis. | | Meta-analysis of 16 Hospital-based or community-based case-control and cohort studies | South East Asia, North America, Europe | Mortality due to all cause, all cancer, UADT cancer, stomach cancer, cervical cancer, IHD, stroke | In random effects, compared to those who did not use SLT, SLT use (snus, tobacco tooth powders, snuff, gutka, khaini, tambaku, qiwam, dohra, kimam, tobacco powder, mawa) was significantly associated with all-cause mortality (OR=1.33; 95% CI: 1.11-1.34), all cancer mortality (OR=1.31; 95% CI: 1.16-1.47), IHD mortality (OR=1.10; 95% CI: 1.04-1.17) and stroke mortality (OR=1.37; 95% CI: 1.24-1.51). Each study was unique in its handling of confounders and a variety of factors (smoking), however, inclusion criteria constrained all included studies to be adjusted for tobacco smoking. There was no obvious evidence of publication bias for any of the outcomes according to the tests for heterogeneity and visual inspection of funnel plots. | **3A** | **Poor** |
| Vidyasagaran, et al. Use of smokeless tobacco and risk of cardiovascular disease: A systematic review and meta-analysis. | | Meta-analysis of 19 studies (cohort studies and case controls) | Global (NA, Asia, Europe) | IHD based on ICD (for 15 of 19 studies) | 18 studies restricted to never tobacco users, two studies included former smokers and adjusted for smoking (did not specify smoking variable). Overall, significantly increased risk of IHD deaths (1.15; 95% CI: 1.01-1.30) and stroke deaths (1.39; 95% CI: 1.29-1.49) was found among ever-users of ST. Heterogeneity was 70% (IHD deaths) and 0% (stroke deaths). | **3A** | **Good** |
| **MORBIDITY** | | | | | | | |
|  | |  |  |  |  |  |  |
| **CANCER** | |  |  |  |  |  |  |
| Asthana, et al. Association of smokeless tobacco use and oral cancer: a systematic global review and meta-analysis. | | Meta-analysis of 37 case-control and cohort studies | Global (SEA, Europe, Americas) | Oral cancer | Ever SLT use (all types) was associated with oral cancer across all WHO regions (OR=3.52; 95% CI: 2.75-4.51), compared to those who did not use smokeless tobacco (adjusted for smoking).   Effect (using random effects model) differed by product: • Oral snuff (OR=4.18; 95% CI: 2.37, 7.38) • Gutkha (OR=8.67; 95% CI: 3.59, 20.95) • Manipuri (OR=3.32; 95% CI: 1.32, 8.36) • Pan tobacco/arecea nut + lime + tobacco: OR=7.18; 95% CI: 5.48, 9.41) • Nasal snuff/dipping: OR=1.20; 95% CI: 0.80, 1.81)  Heterogeneity was significant for all of the above except snuff/dipping (p=0.38). Meta-analysis with the random-effect model in the funnel plot, the observed effect sizes are more or less symmetrically distributed around the combined effect size inferring absence of bias. | **3A** | **Poor** |
| Siddiqi, et al. Global burden of disease due to smokeless tobacco consumption in adults: analysis of data from 113 countries. | | Meta-analysis and comparative risk assessment of 32 cohort and case-control studies | Global (South East Asia, Europe, North America) | Oral, pharyngeal, esophageal cancers | Relationship between SLT use and cancer (compared to non- tobacco users) was significant across all regions for mouth (oral cavity, tongue, and lip) cancers (OR=3.43; 95% CI: 2.26-5.19), pharynx cancer (OR=2.23; 95 % CI: 1.55-3.20) or esophageal cancers (OR=2.17; 95% CI: 1.70-2.78). No tests for publication bias. Most studies adjusted for, but did not exclude, smoking. | **3A** | **Poor** |
| **CARDIOVASCULAR** | |  |  |  |  |  |  |
| Gupta, et al. Association of smokeless tobacco and cerebrovascular accident: a systematic review and meta-analysis of global data | | Meta-analysis of 14 studies | Global (EU, SEA, Americas, EMR) | IHD Mortality, stroke | Among smoking adjusted studies (excluded smokers or adjusted for), across all regions (Europe, US, Southeast Asia, Mediterranean), compared to non-users of SLT, exclusive SLT users (all types) had higher risk of stroke (OR=1.18; 95% CI: 1.04-1.32) and fatal stroke (OR=1.27; 95% CI: 1.15-1.39), but not nonfatal stroke (OR=1.03; 95% CI: 0.91-1.14), compared to non-users. No significant publication bias was found in the included studies. Snuff and nass were not associated with risk of stroke, but chewing tobacco was associated with increased risk of stroke (OR=1.35; 95% CI: 1.20-1.50). No significant publication bias was found in the included studies. Only those studies were included in the analysis that had exclusive SLT users as subjects or which included smokers as well as SLT users but listed separate results for SLT users. Heterogeneity was limited in all regions except the US studies (66%). | **3A** | **Poor** |
| Gupta, et al. Risk of coronary heart disease among smokeless tobacco users: results of systematic review and meta-analysis of global data. | | Meta-analysis, 20 studies from four WHO regions | Global (Europe, EMR, Americas, SEA) | CHD | Using of random effects model, SLT use (snuff, chewing tobacco, Naswar) not significantly associated with CHD (Summary OR=1.05; 95% CI: 0.96-1.15). When removing studies that did not adjust for smoking or did not include exclusive SLT users, risk of CHD did not change (OR=1.05; 95% CI: 0.95-1.16).  Among smoking-adjusted studies, risk of CHD (OR=1.13; 95% CI: 0.92-1.06) among chewing tobacco users was not significant. Risk of CHD among Naswar users was significant (OR=1.30; 95% CI: 1.06-1.54). Heterogeneity was found among Southeast Asia studies for nonfatal CHD (57.2%). Egger’s regression test and Begg’s test indicated presence of publication bias among the included studies. Studies not adjusting for smoking were excluded (no information on smoking definition across studies). | **2A** | **Good** |
| Vidyasagaran, et al. Use of smokeless tobacco and risk of cardiovascular disease: A systematic review and meta-analysis. | | Meta-analysis of 19 studies (cohort studies and case controls) | Global (NA, Asia, Europe) | IHD based on ICD (for 15 of 19 studies) | 18 studies restricted to never tobacco users, two studies included former smokers and adjusted for smoking (did not specify smoking variable). Using random effects model, ever self-reported SLT users (including SLT, chew, dip, snus, snuff) did not show elevated risk of IHD (1.14; 95% CI: 0.92-1.42) or stroke (1.01; 95% CI: 0.90-1.13). Stroke studies were only from Europe. Overall, significantly increased risk of IHD deaths (1.15; 95% CI: 1.01-1.30) and stroke deaths (1.39; 95% CI: 1.29-1.49) was found among ever-users of ST. Heterogeneity was 80% (IHD) and 0% (stroke). | **3A** | **Good** |
| **AMEA** | | | | | | | |
| **MORTALITY** | | | | | | | |
| Etemadi, et al. Hazards of cigarettes, smokeless tobacco and waterpipe in a Middle Eastern Population: a Cohort Study of 50 000 individuals from Iran | | Longitudinal cohort, 50,045 adult participants | Iran | Overall mortality, IHD mortality, CVA mortality, cancer mortality | Compared to self-reported never tobacco users (any type), current self-reported nass chewing was associated with overall mortality (HR=1.16; 95% CI: 1.01-1.34), adjusting for but not excluding cigarette smoking. When looking at current nass use only (who were never smokers) compared to never tobacco use, there was a marginally significant increased risk of mortality (HR=1.17; 95% CI: 1.00-1.36) and cancer mortality (HR=1.40; 95% CI: 1.01-1.95), but not elevated risk of IHD mortality (HR=1.32; 95% CI: 1.05-1.67), CVA mortality (HR=1.06; 95% CI: 0.74-1.53) or respiratory mortality (HR=1.73; 95% CI: 0.94-3.19). However, among former cigarette users/current nass users, there was greater risk of mortality (HR=1.33; 95% CI: 1.11-1.59) and cancer mortality (HR=1.65; 95% CI: 1.13-2.39) compared to never tobacco users. Dual users (current cigarette users/current nass users) also had elevated risk of overall mortality (HR=1.28; 95% CI: 1.00-1.64) and cancer mortality (HR=1.67; 95% CI: 1.02-2.75) but not IHD, CVD or respiratory mortality. | **2B** | **Fair** |
| Gupta, et al. Association of smokeless tobacco and cerebrovascular accident: a systematic review and meta-analysis of global data | | Meta-analysis of 14 studies | Global (EU, SEA, Americas, EMR) | IHD Mortality, stroke | Among smoking adjusted studies (excluded smokers or adjusted for), in South East Asian region, compared to non-users of SLT, exclusive SLT (all types) users had higher risk of fatal stroke (OR=1.35; 95% CI: 1.18-1.51) compared to non SLT-users. No significant publication bias was found in the included studies. | **3A** | **Poor** |
| Sinha, et al. Global burden of all-cause and cause-specific mortality due to smokeless tobacco use: systematic review and meta-analysis. | | Meta-analysis of 16 Hospital-based or community-based case-control and cohort studies | South East Asia, North America, Europe | Mortality due to all cause, all cancer, UADT cancer, stomach cancer, cervical cancer, IHD, stroke | In random effects, compared to those who did not use SLT, SLT in Southeast Asian region was significantly associated with all-cause mortality (OR=1.25; 95% CI: 1.08-1.44), all cancer mortality (OR=1.46; 95% CI: 1.26-1.68), stroke mortality (OR=1.37; 95% CI: 1.14-1.64), but not IHD mortality. Each study was unique in its handling of confounders and a variety of factors (smoking), however, inclusion criteria constrained all included studies to be adjusted for tobacco smoking. There was no obvious evidence of publication bias for any of the outcomes according to the tests for heterogeneity and visual inspection of funnel plots. | **3A** | **Poor** |
| Vidyasagaran, et al. Use of smokeless tobacco and risk of cardiovascular disease: A systematic review and meta-analysis. | | Meta-analysis of 19 studies (cohort studies and case controls) | Global (NA, Asia, Europe) | IHD based on ICD (for 15 of 19 studies) | Studies from Asia did not show increased risk of IHD death (OR=1.05; 95% CI: .076-1.47). Publication bias did not appear substantial based on visual inspection of the funnel plot. | **3A** | **Good** |
| Gajalakshmi, et al. Tobacco chewing and adult mortality: a case-control analysis of 22,000 cases and 429,000 controls, never smoking tobacco and never drinking alcohol. South India. | | Population-based case-control study, 22,460 cases and 429,306 controls | India | All-cause mortality, cancer deaths, respiratory deaths, stroke deaths (verbal autopsy) | Among never smokers, risk of all-cause mortality was greater among ever tobacco chewers (reported by family) than never tobacco chewers, (RR = 1.3; 95% CI: 1.2-1.4); Among never smokers, mortality forever tobacco chewers compared to never chewers was significant for deaths from respiratory diseases combined (RR=1.5; 95% CI: 1.4-1.7), respiratory tuberculosis (RR=1.7; 95% CI: 1.5-1.9), cancers (all sites combined) (RR=1.5; 95% CI: 1.4-1.7) and stroke (RR=1.4; 95% CI:1.2-1.6). | **3B** | **Poor** |
| **MORBIDITY** | | | | | | | |
| **CANCER** | |  |  |  |  |  |  |
| Gholap et al. Ecological analysis to study association between prevalence of smokeless tobacco type and head-and-neck cancer. | | Ecological analysis from population-based cancer registries | India | Head and neck cancer | Betel quid and tobacco use correlated (r = 0.53) with oropharynx cancer incidence. Prevalence of Khaini use correlated with hypopharynx cancer incidence (r = 0.48). Gutka use correlated with mouth cancer incidence (r = 0.54). Oral tobacco correlated with mouth cancer incidence (r = 0.46). Other SLT use correlated for hypopharynx cancer incidence (r = 0.47). Did not account for smoking. | **2C** | **Fair** |
| Nair, et al. Squamous cell carcinoma of the upper aerodigestive tract in exclusive smokers, chewers, and those with no habits | | Cross-sectional analysis of 747 participants with head and neck cancer | India | Pathological-confirmed head and neck squamous cell carcinoma (SCC) | No HR/OR reported. Site distribution revealed patients with chewing as the only habit had oral cancers (most commonly gingivobuccal complex cancers) as the most common site and those with smoking as the only habit had larynx as the most common site. Dual users were excluded. | **2C** | **Poor** |
| Asthana, et al. Association of smokeless tobacco use and oral cancer: a systematic global review and meta-analysis. | | Meta-analysis of 37 case-control and cohort studies | Global (SEA, Europe, Americas) | Oral cancer | Ever SLT use (all types) was associated with oral cancer in Southeast Asia (OR=4.44; 95% CI: 3.51-5.61) and in Eastern Mediterranean (OR=1.28; 95% CI: 1.04-1.56), compared to those who did not use SLT (adjusted for smoking). Heterogeneity was significant in Southeast Asia (p<.001). Meta-analysis with the random-effect model in the funnel plot, the observed effect sizes are more or less symmetrically distributed around the combined effect size inferring absence of bias. | **3A** | **Poor** |
| Khan, et al. Naswar (Smokeless Tobacco) Use and the Risk of Oral Cancer in Pakistan: A Systematic Review With Meta-Analysis | | Meta-analysis of six case control studies | Pakistan | Squamous cell carcinoma arising in the oral cavity or the oropharynx based on ICD-10 classification | Ever use of Naswar (from medical history, interviews, questionnaire) is associated with oral cancer (OR=11.8; 95% CI: 11.4-25.3; I2=67%) compared to never use of Naswar. After excluding one study with small sample size and two for unadjusted results, odds increased (OR=11.4; 95% CI: 12.5-28.2); I2= 0%. Four of six studies adjusted for smoking. | **3A** | **Good** |
| Prasad, et al. Risk of major cancers associated with various forms of tobacco use in India: a systematic review and meta-analysis. | | Meta-analysis of 22 studies | India | Six cancers (oral, oropharynx, esophagus, larynx, hypopharynx, and lung) | *Among those who chewed tobacco, compared to those who did not chew tobacco:* Oral cancer: OR=6.6 (95% CI:5.2-8.4) Larynx: cancer OR=1.42 (95% CI: 0.69-2.90) Lung cancer: OR=2.15 (95% CI: 1.22-3.78) Esophagus cancer: OR=3.46 (95% CI: 1.95-5.72) Oropharynx cancer: not significant Hypopharynx cancer: not significant  Studies of mixed (smoking and chewing tobacco) were excluded if the effects of forms of tobacco use could not be shown separately. | **3A** | **Fair** |
| Quadri, et al. Smokeless tobacco and oral cancer in the Middle East and North Africa: A systematic review and meta-analysis | | Meta-analysis of 3 cross-sectional and 3 case-control articles | Middle east and North Africa | Oral cancer | Three case-control studies revealed a pooled estimate odds ratio of 38.74 (95% CI: 19.50-76.96) of oral cancer among SLT users (Shammah use) compared to non-users. Heterogeneity not significant (I2=0%). Evaluation of bias not possible given number of studies. One study adjusted for smoking, not reported in other two. | **3A** | **Poor** |
| Siddiqi, et al. Global burden of disease due to smokeless tobacco consumption in adults: analysis of data from 113 countries. | | Meta-analysis and comparative risk assessment of 32 cohort and case-control studies | Global (South East Asia, Europe, North America) | Oral, pharyngeal, oesophageal cancers | Relationship between SLT use and cancer (compared to non-tobacco users) was significant for mouth (oral cavity, tongue, and lip) cancers in India (OR=5.12; 95% CI: 3.27-8.02) and Pakistan (OR=8.81; 95% CI: 3.14-24.69). It was significant for pharynx cancer in India (OR=2.60; 95% CI: 1.76-3.85), as well as for oesophageal cancers in India (OR=2.57; 95% CI: 2.20-3.00) and Pakistan (OR=8.20; 95% CI: 1.45-27.47). No tests for publication bias. Most studies adjusted for, but did not exclude, smoking. | **3A** | **Poor** |
| Sinha, D. N., Abdulkader, R. S., & Gupta, P. C. (2016). Smokeless tobacco‐associated cancers: A systematic review and meta‐analysis of Indian studies. International journal of cancer, 138(6), 1368-1379. | | Meta-analysis of 25 studies | India | oral cavity, pharynx, larynx, oesophagus, lung, penis, stomach, uterine cervix, female breast, gall bladder, nasal cavity, prostate gland, urinary bladder, kidney, brain, skin, colon and rectum and extra‐hepatic bile duct and sarcoma, non‐Hodgkin lymphoma (NHL), leukaemia and multiple myeloma | Each study was unique in its handling of confounders and a variety of factors (smoking), however, inclusion criteria constrained all included studies to be adjusted for tobacco smoking. SLT (tambaku, qiwam, dohra, kimam, tobacco powder, mawa and others) was significantly associated with the following: • Oral cancer (OR=5.67; 95% CI: 3.83-8.40) • Pharyngeal cancer (OR=2.69; 95% CI: 2.28-3.17) • Oesophageal cancer (OR=3.17; 95% CI: 2.76-3.63) The I2 value was highest (about 95%) for the meta-analyses of oral and laryngeal cancers indicating the high heterogeneity but was relatively lower for other cancers. Tests of publication bias (Begg-Mazumdar’s test and Egger’s test) were non-significant for all outcomes | **3A** | **Poor** |
| Alharbi, et al. Individual and integrated effects of potential risk factors for oral squamous cell carcinoma: a hospital-based case-control study in Jazan, Saudi Arabia. | | Hospital-based case-control in Saudi Arabia, 70 cases, 140 controls | Saudi Arabia | History-pathologically confirmed squamous cell carcinoma of oral cavity (OSCC) using ICD 10th revision | Patient record-extracted ever-use of shammah (OR=33.01; 95% CI: 3.22-39.88) and shisha (OR=3.96; 95% CI: 0.24-63.38) consumption was significantly associated (P<0.05) with oral squamous cell carcinoma development compared to never use (no control for cigarette use). Combined use of shammah and shisha increased the chance of OSCC by nearly 35 times (OR=35.03; 95 % CI = 11.50 -65.66) compared to never use. While, cigarette if used along with shammah (OR=10.10; 95% CI: 0.50-20.40) or shisha (OR=10.52; 95% CI: 1.03 -33.90) increased the chance of OSCC by nearly 10 times. | **3B** | **Poor** |
| Awan et al. Assessing the Risk of Oral Cancer associated with Gutka and Other Smokeless Tobacco Products: A Case-control Study.B19 | | Hospital-based case-control, 134 cases, and 134 age-matched controls | Pakistan | Histopathologically confirmed diagnosis of oral cancer | Odds of developing oral cancer was higher among self-reported gutka users (OR=5.54; 95% CI: 2.83-10.83; p < 0.001) compared to participants who did not consume gutka. Chewing tobacco users had 5.32 (95% CI: 1.14-24.77; p = 0.033) times higher odds compared to participants who did not chew tobacco. Study says they controlled for confounders, but did not mention which confounders. Participants included those who chewed and smoked tobacco. Not clear how they were handled or how exposure was measured. | **3B** | **Fair** |
| Chang, et al. Tobacco smoking, chewing habits, alcohol drinking and the risk of head and neck cancer in Nepal. | | Hospital-based case control study 549 cases, 601 controls | Nepal | Pathological diagnosis of oral cavity, oropharynx, hypopharynx and larynx cancers | Ever use of tobacco chewing was associated with head and neck cancer (OR=2.39; 95% CI: 1.77-3.23) compared to never use (adjusted for tobacco smoking; duration and frequency). Compared to 0 times per day, chewing tobacco 0-6 was more likely (OR=1.95; 95% CI: 1.37-2.7) to be associated with head and neck cancer, and >6 per day was also more likely (OR=2.91; 95% CI: 2.06-4.12). Compared to 0 years, 0-20 years of chewing was associated with increased risk (OR=1.86; 95% CI: 1.29-2.67) and >20 years of chewing was associated with increased risk (OR=2.92; 95% CI: 2.08-4.11) | **3B** | **Fair** |
| Gupta, et al. Associations between oral hygiene habits, diet, tobacco and alcohol and risk of oral cancer: A case-control study from India | | Hospital-based case-control, 187 cases and 240 controls | India | Histopathologically confirmed diagnosis of squamous cell carcinoma of the oral cavity | Self-reported ever-chewers had elevated odds of oral cancer compared to never chewers (OR=8.51; 95% CI: 4.90-14.77), adjusted for smoking tobacco. A linear dose-response association was observed between oral cancer and chewing tobacco in terms of age at initiation, duration, and frequency of chewing per day (P < 0.001). | **3B** | **Fair** |
| Hassanin, et al. Attribution of oral cancer in the Sudan to Tokomak dipping. | | Hospital-based case-control, 196 (98 cases and 98 controls) | Sudan | Pathology confirmed oral squamous cell carcinoma | Self-reported Tokomak dipping was significantly associated with oral squamous cell carcinoma (OR=3.8; 95% CI: 1.7-8.6) compared to those who did not use Tokomak (controlled, but not did exclude, cigarette smoking). Attributable risk for Tokomak use was 66.9% for males and 93.4% for females and 70.1% overall. | **3B** | **Poor** |
| Kadashetti et al. Analysis of various risk factors affecting potentially malignant disorders and oral cancer patients of Central India. | | Hospital-based case-control study, 100 cases, 100 controls | India | Histopathologically diagnosed oral cancer and potentially malignant disorders | Self-reported tobacco chewers more likely to have oral cancer (OR=2.8; 95% CI: 1.2-7.0) compared to never tobacco chewers. Self-reported tobacco chewers and smokers (dual users) did not have an increased risk of oral cancer (OR=0.7; 95% CI: 0.2-2.6) compared to those who did not chew or smoke tobacco (adjusted only for age and sex). | **3B** | **Fair** |
| Khan, et al. Oral cancer via the bargain bin: The risk of oral cancer associated with a smokeless tobacco product (Naswar). | | Multi-center matched case-control study in Pakistan, 84 cases, 174 controls | Pakistan | Clinical diagnosis of oral cancer | Compared to never users, ever users, current users and past users of self-reported Naswar more likely to have oral cancer (ever: OR=21.0; 95% CI: 6.1-72.1, current: OR=23.4; 95% CI: 6.6-82.1, past: OR=16.4; 95% CI: 4.1-65.4), controlling for smoking. Risk varied by Naswar pack years, compared to 0-10 years, 11-20 years (OR=9.6; 95% CI: 3.6-25.5) and >20 years (OR=8.7 95% CI: 3.3-22.6) had increased risk, controlling for smoking. | **3B** | **Poor** |
| Khan, et al. Smokeless tobacco use and risk of oral cavity cancer | | Hospital-based case control study, 90 cases, 120 controls | Pakistan | Laboratory confirmed primary diagnosis of oral cavity cancer | Odds of oral cancer among self-reported SLT users was 4.71 (95% C: 2.53-8.74) compared to never SLT users (controlling for smoking, but not excluding smokers). Snuff users had highest risk (OR=4.82; 95% CI: 2.37-9.80), followed by betal leaf (OR=4.42; 95% CI: 1.66-11.91) and Supari/Chalia (OR=4.67; 95% CI: 1.14-19.12) compared to never SLT users (adjusted for smoking) | **3B** | **Poor** |
| Merchant, et al. Total, direct, and indirect effects of pan on oral cancer. | | Hospital-based case-control in Pakistan, 79 cases, 143 controls | Pakistan | Biopsy confirmed primary oral squamous cell carcinoma | Self-reported paan without tobacco increased oral cancer risk 7 fold (OR=7.39; 95% CI: 1.01-38.11). The natural indirect effect (oral cancer risk due to oral subcutaneous fibrosis (OSMF) among users of paan without tobacco) was OR=2.48 (95% CI: 0.99-10.44), and the natural direct effect (oral cancer risk due to paan without tobacco in the absence of OSMF) was OR=3.32 (95% CI: 0.68-10.07). | **3B** | **Fair** |
| Mohite, et al. Exposure to Smokeless Form of Tobacco and Risk of Breast Cancer: A Case Control Study from Rural Maharashtra, India | | Hospital-based case-control in India, 217 cases, 217 controls | India | Clinically and histo-pathologically confirmed breast cancer | Compared to those who did not self-report SLT use, those who report exposure to SLT are more likely to have breast cancer (OR=2.35; 95% CI 1.01-5.51); longer duration of >5/day (OR=10.13; 95% CI: 5.41-18.23) and greater frequency of exposure of >10 years (OR=31.13; 95% CI: 11.67-39.82) increased odds of breast cancer (no information on variables controlled for). No information on confounder variables or smoking. | **3B** | **Poor** |
| Nair, et al. Colorectal cancer and its risk factors among patients attending a tertiary care hospital in Southern Karnataka, India. | | Hospital-based case-control study, 100 cases, 200 controls | India | Histological-confirmed colorectal cancer | Compared to non-users, self-reported chewing tobacco was not associated with colorectal cancer (OR=1.53; 95% CI: 0.58-4.00). Smoking was not included in the multivariable analysis, as it was not significantly associated with colorectal cancer. | **3B** | **Poor** |
| Quadri, et al. Oral squamous cell carcinoma and associated risk factors in Jazan, Saudi Arabia: a hospital based case control study | | Hospital case control study 48 cases, 96 controls | Saudi Arabia | Diagnosed and histologically confirmed oral cancer | Controlling for cigarette smoking, self-reported shamma use (SLT) increases odds of oral cancer by 29 times (OR=29.30; 95% CI: 10.33-83.13) compared to non-users. With the interaction model the odds ratio increased significantly for shamma users (OR=37.2; 12.3-113.2) and cigarette smokers (OR=10.5; 2.88-3.11). Khat was observed to have negative effect on the disease occurrence when used along with shamma (OR=0.01; 0.00-0.65). | **3B** | **Fair** |
| Rajbongshi, et al. Evaluation of female breast cancer risk among the betel quid chewer: A bio-statistical assessment in Assam, India. | | Hospital-based matched case-control in India, 100 female cases, 100 female controls | India | Histopathologically confirmed  breast cancer | Self-reported chewing tobacco was associated with greater risk of breast cancer (OR=2.35; 95% CI: 1.3-4.15) compared to those who did not chew tobacco (did not account for smoking). | **3B** | **Poor** |
| Shah, et al. Tobacco chewing and risk of gastric cancer: a case-control study in Yemen. | | Matched case-control study, 210 participants (70 cases, 140 controls) | Yemen | Histologically confirmed gastric cancer | Odds of gastric cancer were 4.4 times higher among those who self-reported chewing tobacco use than non-chewers (OR=4.37; 95% CI: 1.92-9.95). There was no relationship between smoking cigarettes and gastric cancer (OR=1.74; 95% CI: 0.97-3.11). | **3B** | **Fair** |
| Sajad, et al. Indiscriminate Use of Smokeless Tobacco Leading to Oral Cancer at a Young Age; A Case Report with Literature Review on Tobacco. | | Case report, 35 year-old male | India | Squamous cell carcinoma | OSCC in buccal mucosa and the reason for the same was exclusive unilateral tobacco chewing habit, with placement of the tobacco in the right mucobuccal fold. | **4** | **Fair** |
| Mahapatra et al. Risk of oral cancer associated with gutka and other tobacco products: A hospital-based case-control study. | | Hospital-based case-control,134 cases and 268 controls | India | Laboratory-confirmed primary diagnosis of oral cancer according to ICD-10 | Those who self-reported chewing tobacco use were 6.0 (95% CI: 2.6-15.5; P < 0.01) times more likely to get oral cancer as compared to people who did not use chewing tobacco. Results differed by product: snuff: OR=1.0 (95% CI: 0.3-3.0); Gutka: OR=5.1 (95% CI: 2.0-10.3); Supari: OR=11.4 (95% CI: 3.4-38.2); Betel quid: OR=6.4 (95% CI: 2.6-15.5). Controlled for “other tobacco” types, did not exclude other tobacco users/smokers. | **4** | **Poor** |
| Soni, et al. A Study on Oral Cancer and Its Correlation with Tobacco Chewing, Smoking and Alcohol Drinking In Western Rajasthan. | | Case series (no control group), 100 cases | India | Histopathologically diagnosed oral cancer | No details about methods. There were 9 individuals with no habit of smoking or alcohol consumption. Reported significant different in oral cancer among patients who exclusively chewed tobacco than non-users of tobacco. | **4** | **Poor** |
| **CARDIOVASCULAR** | |  |  |  |  |  |  |
| Gupta, et al. Risk of coronary heart disease among smokeless tobacco users: results of systematic review and meta-analysis of global data. | | Meta-analysis, 20 studies from four WHO regions | Global (Europe, EMR, Americas, SEA) | CHD | In South-east Asia region, there was not significant relationship between SLT use and CHD (OR=1.02; 95% CI: 0.86-1.18), which did not change when excluding studies that did not adjust for cigarette smoking (OR=1.00; 95% CI: 0.85-1.15). Risk of non-fatal CHD (OR=1.10; 95% CI: 1.00-1.20) and fatal CHD (OR=1.03; 95% CI: 0.86-1.19) were not significant. In Eastern Mediterranean region, using random effects model, SLT use was associated with CHD (OR=1.41; 95% CI: 1.13-1.69), however there was no significant risk of fatal CHD or non-fatal CHD in the region. Exclusion of studies not adjusting for cigarette smoking did not change the risk.  Egger’s regression test and Begg’s test revealed similar statistically non-significant result, indicating presence of publication bias among the included studies. | **2A** | **Good** |
| Ahwal, et al. A Study to Compare The Cardiovascular Disease (CVD) Risk Associated with Smokeless Tobacco Consumption and Smoking. | | Cross-sectional survey, 30 exclusively SLT consumers, 30 exclusive smokers and 30 non-tobacco consumers | India | Biophysiological measurements of obesity or overweight, hypertension, blood sample-measured impaired fasting blood glucose, diabetes mellitus, dyslipidemia | HDL cholesterol was significantly higher only in self-reported exclusive SLT users (p=0.004); exclusive SLT users had increased odds of dyslipidemia (OR=6.37; 95% CI: 1.4-27.3) and hypertension (OR=6.97; 95% CI: 1.7-28.0) compared to non-tobacco users (no accounting for former smoking). | **2C** | **Poor** |
| Anand, et al. The risk of hypertension and other chronic diseases: Comparing smokeless tobacco with smoking. | | Cross-sectional study, 4,038 respondents | India | Self-reported diabetes, asthma, hypertension | Risk of diabetes and hypertension not statistically significant among self-reported exclusive SLT users compared to exclusive smokers (former smoking not considered). | **2C** | **Fair** |
| Bhatt, et al. Predictors of hypertension among nonpregnant females attending health promotion clinic with special emphasis on smokeless tobacco: A cross-sectional study. | | Cross-sectional study, 314 non-pregnant females who did not use cigarettes | India | Measured systolic BP, diastolic BP | Systolic hypertension was associated with quantity of SLT use (B= 0.389 (SE = 0.131); p= 0.003), but diastolic hypertension was not (B=0.122 (SE=0.087); p= 0.160). Study did not report on whether smoking status was accounted for. | **2C** | **Good** |
| Mishra, et al. Latent coronary artery disease among smokers and smokeless tobacco users: a cross-sectional study. | | Cross-sectional survey, 36 participants with mental and behavioral disorders due to use of tobacco (ICD 10) | India | Latent CHD measured by treatment exercise test | No difference in CHD between those who exclusively smoke and those who use exclusive SLT (determined from clinical assessment). Those with history of dual use were excluded. | **2C** | **Poor** |
| Gupta, et al. Association of smokeless tobacco and cerebrovascular accident: a systematic review and meta-analysis of global data | | Meta-analysis of 14 studies | Global (EU, SEA, Americas, EMR) | IHD Mortality, stroke | Among smoking adjusted studies (excluded smokers or adjusted for), in South East Asian region, compared to non-users of SLT, exclusive SLT (all types) users had higher risk of stroke (OR=1.35; 95% CI: 1.18-1.51) and fatal stroke (OR=1.35; 95% CI: 1.18-1.51) compared to non SLT-users. No significant publication bias was found in the included studies. | **3A** | **Poor** |
| Vidyasagaran, et al. Use of smokeless tobacco and risk of cardiovascular disease: A systematic review and meta-analysis. | | Meta-analysis of 19 studies (cohort studies and case controls) | Global (NA, Asia, Europe) | IHD based on ICD (for 15 of 19 studies) | Studies from Asia showed increased risk of IHD (OR=1.40; 95% CI: 1.01-1.95) among ever SLT users compared to never tobacco users, but not increased risk of IHD death (OR=1.05; 95% CI: .076-1.47). Heterogeneity was 81%. Publication bias did not appear substantial based on visual inspection of the funnel plot. | **3A** | **Good** |
| Behera, et al. Impact of smokeless tobacco products on myocardial infarction and stroke and it’s prognostic significance. | | Prospective cohort study, 423 participants with complaints suggesting of CHD and stroke | India | Self-reported stroke and MI | Odds of stroke was higher for those who self-reported SLT use (OR=3.71; 95% CI: 1.57-9.05 (p=0.002)) compared to those who did not use SLT, excluding current smokers (but not former smokers). Odds of MI were greater for those who reported SLT use (OR=2.34; 95% CI: 1.10-5.40) compared to those who did not use SLT, excluding current smokers (but not former smokers). | **4** | **Poor** |
| **OTHER** |  |  |  |  |  |  |  |
| Anand, et al. The risk of hypertension and other chronic diseases: Comparing smokeless tobacco with smoking. | | Cross-sectional study, 4,038 respondents | India | Self-reported diabetes, asthma, hypertension, chronic lung disease | SLT users had lower risk of chronic lung disease (OR=0.64; 95% CI: 0.45-0.91) compared to smokers. Risk of asthma not statistically significant among self-reported exclusive SLT users compared to exclusive smokers (former smoking not considered). SLT users had lower risk of chronic lung disease (OR=0.64; 95% CI: 0.45-0.91) compared to smokers. | **2C** | **Fair** |
| Ahwal, et al. A Study to Compare The Cardiovascular Disease (CVD) Risk Associated with Smokeless Tobacco Consumption and Smoking. | | Cross-sectional survey, 30 exclusively smokeless tobacco users, 30 exclusive smokers and 30 non-tobacco users | India | Biophysiological measurements of obesity or overweight, hypertension, blood sample-measured impaired fasting blood glucose, diabetes mellitus, dyslipidemia | There was no difference in the prevalence of obesity (BMI>= 25) among SLT users, smokers and non-tobacco users (p=0.393). No accounting for current or former smoking in analysis. | **2C** | **Poor** |
| Mahapatra, et al. Influence of tobacco chewing on oral health: A hospital-based cross-sectional study in Odisha. | | Hospital-based cross-sectional study, 512 participants | India | Oral health from modified WHO Oral Health Assessment Form | Compared to the self-reported nonchewers, exclusive current tobacco chewers (who chewed for at least one year and were non-smokers) had significantly higher odds for gingival bleeding (OR=1.710; 95% CI: 1.2-2.43) as well as periodontal pockets (OR=1.715; 95% CI: 1.19-2.48); tobacco chewing was also associated with higher odds of loss of attachment (OR=2.393; 95% CI: 1.55-3.69) and attrition (OR=2.496; 95% CI: 1.73-3.61) | **2C** | **Fair** |
| Mathew, et al. Effects of Smokeless Tobacco (Slt) On the General and Reproductive Health of Women in Selected Villages of Udupi District, Karnataka. | | Cross-sectional survey, 800 married women | India | Self-reported general and reproductive health | Significant association between self-reported SLT (study does not mention SLT types) use and obstetrical problems (OR=4.1; 95% CI: 2.54-6.64) and neonatal problems (OR=2.78; 95% CI: 1.59-4.87) compared to non-SLT-users; no relationship between SLT users and gastrointestinal (OR=2.95; 95% CI: 0.92-9.48) or urinary problems (OR=21.15; 95% CI: 0.69-6.64). No mention of smoking in analyses. | **2C** | **Poor** |
| **US** | | | | | | | |
| **MORTALITY** | | | | | | | |
| Fisher, et al. Smokeless tobacco mortality risks: an analysis of two contemporary nationally representative longitudinal mortality studies. | | Pooled study of two longitudinal cohort studies (210,090 participants and 154,286 participants) | US | All-cause and disease-specific (cancer, cardiovascular) mortality | No excess mortality risk among exclusive SLT users (who were never smokers) compared to those who never used tobacco (NHIS: HR=1.03; 95% CI: .83-1.29; NLMS: HR=0.82; 95% CI: 0.59-1.13). Dual users (current smokers/current SLT users) had similar excess risk of mortality (NHIS: HR=2.21; 95 %CI: 1.50-3.26; NLMS: HR=2.14; 95% CI: 1.27-3.59) to exclusive smokers (NHIS: HR=2.10; 95% CI: 1.99-2.22; NLMS: HR=1.88; 95% CI: 1.75-2.02), compared to never tobacco users. Exclusive SLT users (who were never smokers) were no more likely to die from malignant neoplasms of Trachea, bronchus, and lung (HR=2.98; 95% CI: 0.91-9.76), digestive organs (HR=1.01; 95% CI: 0.32-3.20), esophagus (HR=2.44; 95% CI: .31-19.1), pancreas (HR=1.36; 95% CI: .19-9.98) and genitourinary system (HR=0.51; 95% CI: .007-3.78) compared with never tobacco users. Exclusive, SLT users (who were never smokers) were no more likely to die of heart disease (NLMS: HR=1.07; 95% CI:0.65-1.75; NHIS: HR=1.20; 95% CI: 0.91-1.58), heart failure (NLMS: HR=1.13; 95% CI:0.28-4.62; NHIS: HR=2.75; 95% CI: 1.55-4.89) or IHD (NLMS: HR=0.95; 95% CI:0.49-1.83; NHIS: HR=1.06; 95% CI: 0.75-1.49) compared with never tobacco users. | **2A** | **Good** |
| Inoue-Choi, et al. Contemporary associations of exclusive cigarette, cigar, pipe, and smokeless tobacco use with overall and cause-specific mortality in the United States. | | Population-based cohort study, 65,335 individuals | US | Verified mortality, cancer-specific mortality | Current self-reported SLT use (who were never smokers) was associated with a higher mortality risk (HR=1.36; 95% CI: 1.17-1.59) compared to never use of tobacco, with higher risks observed among daily users (HR= 1.41; 95% CI: 1.20-1.66) but not nondaily users. Compared to never use of tobacco, current SLT use (who were never smokers) was associated with CHD mortality (HR=1.63; 95% CI: 1.27-2.09), cancer mortality (HR=1.48; 95% CI: 1.04-2.12) and smoking-related cancer mortality (HR=1.76; 95% CI: 1.07-2.90), but not chronic lower respiratory disease mortality or cerebrovascular disease mortality. | **2B** | **Good** |
| Rodu, et al. Mortality among male smokers and smokeless tobacco users in the USA. | | Cohort study, 46,104 men aged 40-79 years with 28 years of follow up | US | Mortality from all causes (heart diseases, malignant neoplasms, and two mutually exclusive categories: smoking-related and other diseases) | Younger (40-59 years) exclusive ST users (chewing tobacco and/or snuff) who never smoked cigarettes had significantly increased, all-cause mortality (HR=1.44; 95% CI: 1.12-1.84) compared to those who never used ST or cigarettes. Older (60-79 years) exclusive ST users who never smoked cigarettes did not have a statistically significant increased risk of all-cause mortality. Exclusive current ST users who were never cigarette smokers did not have significant elevations in mortality from any other diseases, with the exception of all other causes in younger age group (HR=1.68; 95% CI: 1.11-2.54), compared to those who never used cigarettes or ST. Former cigarette smokers who currently used ST had increased mortality for both younger and older men, compared to those who never used ST or cigarettes. | **2B** | **Good** |
| Timberlake, et al. A longitudinal study of smokeless tobacco use and mortality in the United States. | | Longitudinal cohort, 349,282 participants with no cigarette smoking history | United States | Mortality from all causes, all cancers, coronary heart disease, cerebrovascular disease and digestive system cancers | Individuals who had ever smoked cigarettes, cigars or pipes were excluded from all analyses. No significant association between current self-reported SLT (snuff or chewing tobacco) use and all-cause mortality (HR=1.01; 95% CI: 0.93-1.10), all cancers (HR=0.99; 95% CI: 0.82-1.21) or cerebrovascular disease (HR=0.92; 95% CI: 0.67-1.27) compared to never tobacco users. There was a significant risk of death from CHD (HR=1.25; 95% CI: 1.05-1.46) among current self-reported SLT users compared to never tobacco users. | **2B** | **Fair** |
| Sinha, et al. Global burden of all-cause and cause-specific mortality due to smokeless tobacco use: systematic review and meta-analysis. | | Meta-analysis of 16 Hospital-based or community-based case-control and cohort studies | South East Asia, North America, Europe | Mortality due to all cause, all cancer, UADT cancer, stomach cancer, cervical cancer, IHD, stroke | In random effects, compared to those who did not use SLT, SLT in American region was significantly associated with all-cause mortality (OR=1.17; 95% CI: 1.12-1.22), all cancer mortality (OR=1.14; 95% CI: 1.01-1.29). There was also a significantly elevated risk of stroke mortality (OR=1.44; 95% CI: 1.30-1.59), and risk of IHD mortality (OR=1.16; 95% CI: 1.05-1.28). Each study was unique in its handling of confounders and a variety of factors (smoking), however, inclusion criteria constrained all included studies to be adjusted for tobacco smoking. There was no obvious evidence of publication bias for any of the outcomes according to the tests for heterogeneity and visual inspection of funnel plots. | **3A** | **Poor** |
| Vidyasagaran, et al. Use of smokeless tobacco and risk of cardiovascular disease: A systematic review and meta-analysis. | | Meta-analysis of 19 studies (cohort studies and case controls) | Global (NA, Asia, Europe) | IHD based on ICD (for 15 of 19 studies) | Studies from North America did not show increased risk of IHD death (OR=1.03; 95% CI: 0.83-1.27) among ever SLT users compared to never tobacco (any type) users, but they did have an increased risk of stroke death (OR=1.42; 95% CI: 1.29-1.57). Heterogeneity was 74 % and 0%, respectively. Publication bias did not appear substantial based on visual inspection of the funnel plot. | **3A** | **Good** |
| **MORBIDITY** | | | | | | | |
| **CANCER** |  |  |  |  |  |  |  |
| Asthana, et al. Association of smokeless tobacco use and oral cancer: a systematic global review and meta-analysis. | | Meta-analysis of 37 case-control and cohort studies | Global (SEA, Europe, Americas) | Oral cancer | Ever SLT use (all types) was not associated with oral cancer in America (OR=4.72; 95% CI: 0.66-33.62), compared to those who did not use SLT (adjusted for smoking). Heterogeneity was significant for America (p=<.001). Meta-analysis with the random-effect model in the funnel plot, the observed effect sizes are more or less symmetrically distributed around the combined effect size inferring absence of bias. | **3A** | **Poor** |
| Siddiqi, et al. Global burden of disease due to smokeless tobacco consumption in adults: analysis of data from 113 countries. | | Meta-analysis and comparative risk assessment of 32 cohort and case-control studies | Global (South East Asia, Europe, North America) | Oral, pharyngeal, oesophageal cancers | Relationship between SLT use and cancer (compared to non-tobacco users) was not significant in North America for mouth (oral cavity, tongue, and lip) cancers (OR=0.95; 95% CI: 0.7-1.28), pharynx cancer (single study) (OR=1.59; 95% CI: 0.84 -3.01) or oesophageal cancers (single study) (OR=1.20; 95% CI: 0.10-14.40). No tests for publication bias. Most studies adjusted for, but did not exclude, smoking. | **3A** | **Poor** |
| Wyss et al. Smokeless tobacco use and the risk of head and neck cancer: pooled analysis of US studies in the INHANCE consortium. | | Pooled analysis of 11 case-control studies, 6,772 cases and 8,375 controls | US | ICD-10 determined oral, pharyngeal, and laryngeal cancers | Among never smokers, odds for SLT use and head and neck cancer (HNC) were 1.71 (95% CI: 1.08-2.70) for ever users of snuff compared with never users; odds was 1.20 (95% CI: 0.81-1.77) for ever tobacco chewers compared with never chewers, but analyses restricted to cancers of the oral cavity showed a stronger association of chewing tobacco (OR=1.81; 95% CI: 1.04, 3.17). HNC risk increased with increasing duration of snuff use (Ptrend = 0.007). | **3A** | **Poor** |
| **CARDIOVASCULAR** | |  |  |  |  |  |  |
| Gupta, et al. Risk of coronary heart disease among smokeless tobacco users: results of systematic review and meta-analysis of global data. | | Meta-analysis, 20 studies from four WHO regions | Global (Europe, EMR, Americas, SEA) | CHD | In America, using random effects model, SLT use was not significantly associated with CHD (OR=1.04; 95% CI: 0.83-1.24). Risk did not change when excluding studies that did not adjust for cigarette smoking. No non-fatal risk of CHD reported. Risk of fatal CHD was the same as risk of CHD, and not significant. Egger’s regression test and Begg’s test revealed similar statistically non-significant result, indicating presence of publication bias among the included studies. | **2A** | **Good** |
| Obisesan, et al. Association between Non-Cigarette/Smokeless Tobacco and Hypertension in the National Health Interview Survey: A Pseudo-Panel Analysis. | | Cross-sectional 13, 086 participants | US | Self-reported hypertension | Odds of hypertension diagnosis for those who responded ‘yes’ to the use of non-cigarette tobacco was 0.88 times lower (95% CI: 0.7907-0.9896) than those who responded no (adjusted for years of smoking). | **2C** | **Fair** |
| Gupta, et al. Association of smokeless tobacco and cerebrovascular accident: a systematic review and meta-analysis of global data | | Meta-analysis of 14 studies | Global (EU, SEA, Americas, EMR) | IHD Mortality, stroke | Among smoking adjusted studies (excluded smokers or adjusted for), in the Americas region, compared to non-users of SLT, exclusive SLT (all types) users did not have higher risk of stroke (OR=1.21; 95% CI: 0.90-1.51) or fatal stroke (OR=1.21; 95% CI: 0.90-1.51) compared to non SLT-users. No significant publication bias was found in the included studies. | **3A** | **Poor** |
| Rostron, et al. Smokeless tobacco use and circulatory disease risk: a systematic review and meta-analysis. | | Meta-analysis of 24 studies (17 cohort studies, five case control studies, 1 cross-sectional survey), 7 in the US | Europe, US | Ischemic heart disease (IHD), stroke | Using random effects model, in the US, there was an elevated risk of IHD (RR=1.17; 95% CI: 1.08-1.27) and stroke (RR=1.28; 95% CI: 1.01-1.62) among SLT users (including snuff and chewing tobacco) compared with non-users (regardless of whether individual studies accounted for cigarette smoking status). In most cases, heterogeneity across study estimates within country was limited, although differences were observed for estimates of stroke risk for US SLT users (I2=72.0%). Study quality was generally good, with 12 of the 17 cohort studies, both of the pooled analyses, and three of the five case-control studies receiving at least seven stars on the Newcastle-Ottawa scale. | **3A** | **Good** |
| **OTHER** | | |  |  |  |  |  |
| Hernandez, et al. Relationships among chewing tobacco, cigarette smoking, and chronic health conditions in males 18-44 years of age. | | Cross-sectional survey, 4,930 males 18-44 years old | US | Self-reported chronic health conditions (hypertension, heart attack, CHD, stroke, asthma, skin cancer, other types of cancer, COPD, arthritis, depressive disorder, kidney disease, and diabetes) | Compared to those who reported no tobacco use (former or never smokers who did not chew tobacco), participants who only chewed tobacco (chewed every day or somedays and were either former or never smokers) were 1.49 (95% CI: 1.23-1.81) more likely, those who only smoked (current smokers who did not chew tobacco) were 1.34 (95% CI: 1.13-1.61) more likely and those who both chewed tobacco and smoked (current smokers who chewed tobacco) were 1.95 (95% CI: 1.46-2.59) more likely to report at least one chronic health condition. | **2C** | **Poor** |
| King, et al. Tobacco product use and mental health status among young adults. | | Cross-sectional study, 2,370 college students | US | Self-reported depression (Center for Epidemiological Studies Depression Iowa Short Form), stress (Cohen’s 10-item Perceived Stress Scale), mental health diagnosis | Self-reported past 30-day use of SLT (verve, chewing tobacco, moist or dry snuff (dip), snus, and dissolvables) use was not associated with mental health diagnosis (AOR=1.16; 95% CI: 0.64-2.10; p =0.594) or depression (AOR=0.98; 95% CI: 0.95-1.00) compared to never-tobacco use, controlling for past 30-day cigarette use. SLT and cigar use were not related to stress scale score. Higher stress scale score was associated with increased odds for e-cigarette (AOR=1.03; 95% CI:1.00- 1.05), waterpipe (AOR=1.04; 95% CI:1.01-1.06), cigarette (AOR=1.02; 95% CI:1.00, 1.04), and any tobacco use (AOR=1.02; 95% CI:1.01, 1.04) | **2C** | **Fair** |
| **EUROPE** | | | | | | | |
| **MORTALITY** | | | | | | | |
| Araghi, et al. Smokeless tobacco (snus) use and colorectal cancer incidence and survival: Results from nine pooled cohorts. | | Pooled nine prospective cohort studies, 417,872 male participants | Sweden | All-cause mortality, colorectal cancer | Compared to self-reported never-tobacco users (never any type), no significant relationship between exclusive current snus use and all-cause mortality (HR=1.16; 95% CI: 0.89, 1.50). | **2A** | **Fair** |
| Wilson, et al. Snus use, smoking and survival among prostate cancer patients. International journal of cancer. | | Nested prospective cohort, 9,582 men with prostate cancer | Sweden | Prostate cancer mortality, overall mortality | Exclusive snus users (ever users of snus only, non-smokers) had increased risk of overall mortality (HR=1.19; 95% CI: 1.04-1.37) compared to those who did not use tobacco (never users of any tobacco). Both snus and smokers (ever users of both snus and smoking, either concurrently or sequentially) also had increased risk of overall mortality (OR=1.17; 95% CI: 1.06-1.28). In the full cohort, compared to never tobacco users (any type), exclusive snus users (ever use of snus only, never smokers) were at an increased risk of prostate cancer-specific mortality (HR=1.24; 95% CI: 1.03-1.49), but there was no statistically significant risk among those who used both snus and cigarettes (HR=1.08; 95% CI: 0.95-1.23). | **2B** | **Fair** |
| Gupta, et al. Association of smokeless tobacco and cerebrovascular accident: a systematic review and meta-analysis of global data | | Meta-analysis of 14 studies | Global (EU, SEA, Americas, EMR) | IHD Mortality, stroke | Among smoking adjusted studies (excluded smokers or adjusted for), in Europe region, compared to non-users of SLT, exclusive SLT (all types) users did not have higher risk of fatal stroke (OR=1.30; 95% CI: 0.96-1.63) compared to non SLT-users. No significant publication bias was found in the included studies. | **3A** | **Poor** |
| Sinha, et al. Global burden of all-cause and cause-specific mortality due to smokeless tobacco use: systematic review and meta-analysis. | | Meta-analysis of 16 Hospital-based or community-based case-control and cohort studies | South Rast Asia, North America, Europe | Mortality due to all cause, all cancer, UADT cancer, stomach cancer, cervical cancer, IHD, stroke | In random effects, compared to those who did not use SLT, SLT in Europe was not significantly associated with all-cause mortality or all cancer mortality. There was an elevated risk of IHD mortality (OR=1.16; 95% CI: 1.05-1.28), but not stroke mortality. Each study was unique in its handling of confounders and a variety of factors (smoking), however, inclusion criteria constrained all included studies to be adjusted for tobacco smoking. There was no obvious evidence of publication bias for any of the outcomes according to the tests for heterogeneity and visual inspection of funnel plots. | **3A** | **Poor** |
| Vidyasagaran, et al. Use of smokeless tobacco and risk of cardiovascular disease: A systematic review and meta-analysis. | | Meta-analysis of 19 studies (9 cohorts, 11 case control studies), 10 from Sweden, 3 from North America, 6 from Asia | Global (NA, Asia, Europe) | IHD based on ICD (for 15 of 19 studies) | Studies from Europe showed risk of IHD deaths was elevated (OR=1.38; 95% CI: 1.13-1.67), as was risk of stroke death (OR=1.28; 95% CI: 0.98-1.68). Publication bias did not appear substantial based on visual inspection of the funnel plot. Heterogeneity was 39% (IHD deaths) and 0% (stroke deaths). | **3A** | **Good** |
| **MORBIDITY** | | | | | | | |
| **CANCER** |  |  |  |  |  |  |  |
| Araghi, et al. Smokeless tobacco (snus) use and colorectal cancer incidence and survival: Results from nine pooled cohorts. | | Pooled nine prospective cohort studies, 417,872 male participants | Sweden | Colorectal cancer, a--cause mortality, cancer-specific mortality | Compared to self-reported never-tobacco users (any type), no significant association between exclusive self-reported ever snus users and colorectal cancer (HR= 1.22; 95% CI: 0.91, 1.64), no association between former exclusive snus users and colorectal cancer (HR = 1.12; 95% CI: 0.75, 1.67); No association between current exclusive snus users and colon cancer (HR=1.02; 95% CI: 0.81-1.29); Exclusive ever snus users were at increased risk of rectal cancer (HR = 1.29; 95% CI: 1.02-1.30), an increase confined to current users (HR = 1.38; 95% CI: 1.07-1.77). There was no evidence of any dose-response association between snus use and risk of colorectal cancer in relation to either amount or duration of snus use (amount of snus use (cans/week), <4: (HR = 1.19; 95% CI: 0.89-1.58) / 4-6:(HR = 0.77; 95% CI: 0.55-1.07) / ≥7: (HR = 1.36; 95% CI: 1.04-1.78) -duration of snus use (years),<15: (HR = 1.25; 95% CI: 0.91-1.70) / ≥15: (HR =1.04; 95% CI: 0.84-1.29)) | **2A** | **Fair** |
| Araghi, et al. Use of moist oral snuff (snus) and pancreatic cancer: Pooled analysis of nine prospective observational studies. | | Meta-analysis of 9 prospective cohort studies 418,488 total participants | Sweden | Pancreatic cancer (ICD-7 and ICD-10) | Compared to self-reported never-snus use, current snus use was not associated with risk of pancreatic cancer (HR= 0.96; 95% CI: 0.83-1.11), after adjusting for smoking (never, former and current). Moderate degree of heterogeneity between studies observed (I2 statistics 63%). | **2A** | **Fair** |
| Asthana, et al. Association of smokeless tobacco use and oral cancer: a systematic global review and meta-analysis. | | Meta-analysis of 37 case-control and cohort studies | Global (SEA, Europe, Americas) | Oral cancer | Ever SLT use (all types) was not associated with oral cancer in Europe (OR=0.86; 95% CI: 0.58-1.29), compared to those who did not use SLT (adjusted for smoking). There was no heterogeneity found among studies assessing snus (p= 0.47). Heterogeneity was not significant for Europe (p=.88). Meta-analysis with the random-effect model in the funnel plot, the observed effect sizes are more or less symmetrically distributed around the combined effect size inferring absence of bias. | **3A** | **Poor** |
| Siddiqi, et al. Global burden of disease due to smokeless tobacco consumption in adults: analysis of data from 113 countries. | | Meta-analysis and comparative risk assessment of 32 cohort and case-control studies | Global (South East Asia, Europe, North America) | Oral, pharyngeal, oesophageal cancers | Relationship between SLT use and cancer (compared to no-tobacco users): For mouth (oral cavity, tongue, and lip) cancers, there was no significant association in Sweden (OR=0.92; 95% CI: 0.68 -1.25) or Norway (single study; OR=1.10; 95% CI: 0.5 -2.42). For pharynx cancer, there was no increased risk in Sweden (OR=1.45; 95% CI: 0.3-6.21). For oesophageal cancers, there was an increased risk in Norway (single study; OR=1.40; 95% CI: 1.61 -3.21) and Sweden (OR=1.26; 95% CI: 1.02 -1.56). No tests for publication bias. Most studies adjusted for, but did not exclude, smoking. | **3A** | **Poor** |
| **CARDIOVASCULAR** | |  |  |  |  |  |  |
| Gupta, et al. Risk of coronary heart disease among smokeless tobacco users: results of systematic review and meta-analysis of global data. | | Meta-analysis, 20 studies from four WHO regions | Global (Europe, EMR, Americas, SEA) | CHD | In Europe, using random effects model, SLT use was not significantly associated with CHD (OR=0.95; 95% CI: 0.86-1.04) in smoking-adjusted studies. No significant risk of non-fatal CHD, but there was increased risk of fatal CHD (OR=1.30; 95% CI: 1.14-1.47). Egger’s regression test and Begg’s test revealed similar statistically non-significant result, indicating presence of publication bias among the included studies. | **2A** | **Good** |
| Gupta, et al. Association of smokeless tobacco and cerebrovascular accident: a systematic review and meta-analysis of global data | | Meta-analysis of 14 studies | Global (EU, SEA, Americas, EMR) | IHD Mortality, stroke | Among smoking adjusted studies (excluded smokers or adjusted for), in Europe region, compared to non-users of SLT, exclusive SLT (all types) users did not have higher risk of stroke (OR=1.04; 95% CI: 0.94-1.15) or fatal stroke (OR=1.30; 95% CI: CHD-1.63) compared to non SLT-users. No significant publication bias was found in the included studies. | **3A** | **Poor** |
| Rostron, et al. Smokeless tobacco use and circulatory disease risk: a systematic review and meta-analysis. | | Meta-analysis of 24 studies (17 cohort studies, five case control studies, 1 cross-sectional survey), 7 in the US | Europe, US | Ischemic heart disease (IHD), stroke | Using random effects model, in Sweden, current snus use (who were never smokers) was not associated with IHD (RR=1.04; 95% CI: 0.93-1.16) or stroke (RR=1.04; 95% CI: 0.92-1.17) compared to never tobacco users. In most cases, heterogeneity across study estimates within country was limited. Study quality was generally good, with 12 of the 17 cohort studies, both of the pooled analyses, and three of the five case-control studies receiving at least seven stars on the Newcastle-Ottawa scale. | **3A** | **Good** |
| Vidyasagaran, et al. Use of smokeless tobacco and risk of cardiovascular disease: A systematic review and meta-analysis. | | Meta-analysis of 19 studies (9 cohorts, 11 case control studies), 10 from Sweden, 3 from North America, 6 from Asia | Global (NA, Asia, Europe) | IHD based on ICD (for 15 of 19 studies) | Studies from Europe showed no significant risk of IHD (OR=0.91; 95% CI: 0.83-1.01) among ever SLT users compared to never tobacco users. Risk of stroke was not significant in Europe among ever ST users compared to never ST users (OR=1.01; 95% CI: .90-1.13). Publication bias did not appear substantial based on visual inspection of the funnel plot. Heterogeneity was 0% (IHD and stroke) | **3A** | **Good** |
| **OTHER** |  |  |  |  |  |  |  |
| Rauwolf et al. The influence of smoking and smokeless tobacco on the progression, severity and treatment outcome in alcohol-dependent individuals. | | Hospital-based prospective cohort, 347 alcohol-dependent participants | Sweden | Self-reported alcohol dependence | No significant differences between the four groups regarding total abstinence (i.e. no alcohol consumption after end of treatment), days of alcohol consumption the last 30 days or grams of pure alcohol per week. | **2B** | **Fair** |
| Yang, et al. Moist smokeless tobacco (Snus) use and risk of Parkinson’s disease. | | Meta-analysis of prospective cohort studies, 348 601 men | Sweden | Parkinson’s | Among never cigarette smokers, self-reported ever-snus users had 60% lower Parkinson’s disease risk compared with never-snus users (pooled HR=0.41; 95% CI: 0.28-0.61). Dose-response relationships indicate moderate-heavy amount (pooled HR=0.41; 95% CI: 0.19-0.90) and long-term current-snus users (pooled HR 0.44; 95% CI 0.24-0.83) had the lowest Parkinson’s disease risk. There was no observed heterogeneity (I2 = 0-35%). | **2A** | **Good** |
| Gudnadóttir, et al. An investigation on the use of snus and its association with respiratory and sleep-related symptoms: a cross-sectional population study. | | Cross-sectional population survey, 16,082 participants | Sweden | Self-reported asthma, respiratory symptoms (chronic bronchitis, chronic rhinosinusitis) and sleep-related problems | Among those who have never smoked, compared to self-reported never tobacco users (of any type), current snus users had increased risk of asthma (OR=1.49; 95% CI: 1.20-1.85), chronic bronchitis (OR=1.47; 95% CI: 1.21-1.78) and chronic rhinosinusitis (OR=1.37; 95% CI: 1.11-1.70). Snus use was also associated with asthmatic symptoms, allergic rhinitis, and sleeping problems. | **2C** | **Good** |
